# Bridging traditional Chinese medicine and Alzheimer’s disease: the pivotal role of gut microbiota in multitarget therapeutic mechanisms

**DOI:** 10.3389/fphar.2025.1630205

**Published:** 2025-06-27

**Authors:** Weidong Wu, Tianwei Meng, Lichao Han, Fangfang Jin, Pengfei Han, Yanyan Zhou

**Affiliations:** ^1^ Key Laboratory of Basic Theory of Chinese Medicine, Heilongjiang University of Chinese Medicine, Harbin, China; ^2^ Department of Internal Medicine, Heilongjiang University of Chinese Medicine, Harbin, China; ^3^ Medical Laboratory Center, China Academy of Chinese Medical Sciences, Beijing, China; ^4^ Science and Education Department, Zhangjiakou First Hospital, Zhangjiakou, China

**Keywords:** gut microbiota, traditional Chinese medicine, Alzheimer’s disease, metabolism, interaction, mechanisms

## Abstract

Microbiota-gut-brain axis communication represents another crucial pathway in the pathogenesis of Alzheimer’s disease (AD), whereby gut microbiota significantly impacts AD pathology by modulating immune, metabolic, digestive, and neurological functions. Although research on treating AD through gut microbiota interventions is advancing, substantial breakthroughs remain limited. Given AD’s complex pathological mechanisms, Traditional Chinese Medicine (TCM) presents a clear advantage with its multi-target effects. During the processes of TCM intake, absorption, and therapeutic action, the gut microbiota serves both as a mediator and as a therapeutic target. However, the mechanisms by which TCM interacts with gut microbiota to exert beneficial effects on AD remain largely unclarified. Here, we review the mechanisms through which TCM may intervene in AD from the perspective of gut microbiota, examining the potential mechanisms and clinical application prospects of Chinese herbal medicine in regulating the gut microbiome. This provides a novel theoretical foundation and methodological support for further research into herbal therapies for AD.

## Graphical Abstract

**Figure:**
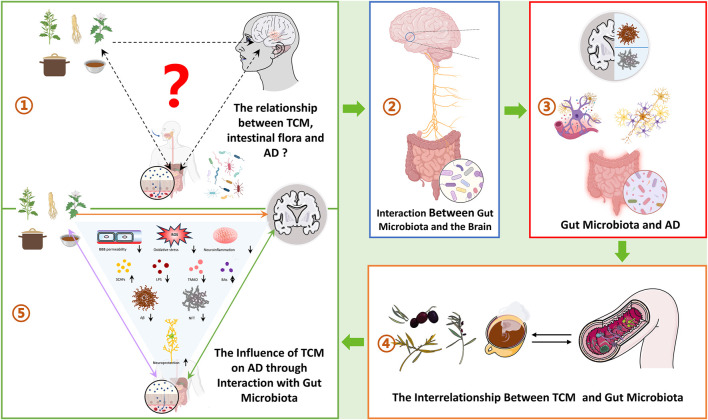

## 1 Introduction

Alzheimer’s disease (AD) is a neurodegenerative condition marked by a gradual deterioration in memory and cognitive abilities. The increasing prevalence of AD presents a significant burden on patients, families, and society. Despite extensive research, the precise pathological mechanisms underlying AD remain unclear. However, the emerging understanding of the bidirectional regulatory relationship between the gut microbiome and the brain has highlighted the link between gut dysbiosis and AD (Liang et al., 2024).

The gut microbiota, a critical component of the human intestinal system, comprises thousands of microorganisms, including bacteria, fungi, viruses, and other microbes. Due to its vast diversity and rich genetic content, it is often referred to as the “second genome” (Lynch and Pedersen, 2016; Zhu et al., 2010). These microorganisms form a highly complex ecosystem in the gut, co-evolving and interacting with the host, and playing an essential role in health and disease. They are involved in key physiological processes such as digestion, immune regulation, metabolic control, and brain function (Jandhyala et al., 2015; Wu and Wu, 2012; Fan and Pedersen, 2021). Especially, gut microorganisms affect brain function through various pathways, including the vagus nerve, metabolites (e.g., short-chain fatty acids), LPS involvement, amyloid production, inflammation and the secretion of endocrine hormones. These mechanisms regulate brain development, anxiety, depression, stress responses, pain, and cognitive functions within the central nervous system (Diaz Heijtz et al., 2011; Vicentini et al., 2021).

The structure and function of the gut microbiota are influenced by numerous factors, including genetic predisposition, lifestyle, diet, medication use, and environmental conditions (Ursell et al., 2012). Notably, aging is a significant factor in gut microbiota changes. By modulating the gut environment, aging-related changes can be improved, benefiting age-associated diseases such as AD (Du et al., 2021; Hong et al., 2022). Current evidence suggests that gut dysbiosis plays a pivotal role in the pathogenesis of AD. Alterations in the abundance and composition of gut microbiota can trigger a range of complex neurological and metabolic disorders through the gut-brain axis, including imbalances in neurotransmitters, metabolites, hormones, immune responses, and barrier functions (Cryan et al., 2019). These alterations ultimately promote neuroinflammation, amyloid-beta deposition, and the formation of neurofibrillary tangles (Dumitrescu et al., 2018; Gao et al., 2019; Wolozin and Ivanov, 2019). Additionally, bacterial-derived amyloid proteins and their interactions with host proteins are potential driving factors in AD pathology, further underscoring the importance of studying the gut microbiota in AD development. Studies have also shown that fecal microbiota transplantation from AD patients to healthy rats induces AD-like symptoms, providing compelling evidence of a causal relationship between the gut microbiota and AD (Grabrucker et al., 2023). Thus, exploring ways to modulate the gut microbiota or leveraging it to improve AD presents a promising research direction.

In this context, traditional Chinese medicine (TCM) emerges as a promising therapeutic strategy. TCM is a holistic medical system that employs natural substances, primarily herbs, and various practices to restore balance and enhance the body’s innate healing capacity. TCM interventions, often administered orally (e.g., decoctions), exert their therapeutic effects by modulating multiple physiological functions and pathological processes. Recently, TCM has gained significant attention as a potential treatment for AD due to its rich natural plant components and its broad spectrum of action targets. It offers new possibilities for drug discovery and research (Sun Z. K. et al., 2013). TCM has been shown to influence gut microbiota composition and improve gut health, which in turn affects neuroinflammation and brain function. For instance, a recent clinical trial demonstrated that the Bushen Yinao pill, a TCM compound formulation, when combined with conventional therapy, significantly modulates gut microbiota composition (increasing beneficial and decreasing harmful bacteria), reduces key inflammatory markers (IL-6 and TNF-α), and improves cognitive function in elderly patients with AD (Wang et al., 2025). Moreover, several studies indicate that Chinese herbal treatments can affect the gut microbiome’s makeup, as well as regulate digestion, immune response, metabolism, and brain function, thereby influencing the progression of various diseases (Chen et al., 2025; Iqbal et al., 2025; Wang H. et al., 2024). Herbal medicines such as *Poria cocos* (Fuling) (Sun et al., 2021), *Rheum tanguticum* (Gao et al., 2021), and *Gastrodia elata* (Huang et al., 2023) have demonstrated significant therapeutic potential. Furthermore, individualized TCM treatment based on syndrome differentiation can alleviate symptoms commonly associated with AD, such as fatigue, irritability, poor appetite, and depression (Lee J. et al., 2022). However, the mechanisms underlying the role of Chinese botanical drugs in gut microbiota modulation remain inadequately understood. Traditionally, Chinese herbal medicines are administered in decoctions, where the active ingredients interact directly with the gut microbiota to regulate its structure and metabolic products. In turn, gut microbes can metabolize these herbal components into secondary metabolites with potent pharmacological effects (Liu et al., 2022a). Consequently, research on the metabolism, absorption, and function of Chinese medicine in relation to the gut microbiota is rapidly advancing. Despite growing interest in the gut-brain axis, critical gaps persist in understanding how TCM orchestrates multitarget AD therapies through microbiota crosstalk. Specifically, there are the following problems: Mechanistic ambiguity: How do TCM phytochemicals dynamically reshape gut microbial ecosystems to concurrently regulate neuroinflammation, amyloidogenesis, and synaptic plasticity? Bidirectional causality: Does TCM primarily drive microbiota restructuring to alleviate AD pathology, or do microbial metabolites enhance TCM bioavailability to exert central effects?Translational barriers: What specific microbial taxa and metabolic pathways serve as actionable targets for standardizing TCM-derived anti-AD interventions?

This review examines the mechanisms by which Chinese medicine intervenes in AD through the gut microbiota. It also explores the role of the gut microbiota in AD and the potential mechanisms by which Chinese botanical drugs can modulate the microbiota, highlighting their clinical application prospects.

## 2 Methods and literature search strategy

A comprehensive literature search was performed using the PubMed, Web of Science, and Google Scholar databases. The search strategy incorporated a combination of keywords such as “gut microbiota”, “gut microbiome”, “Alzheimer’s disease”, “cognitive decline”, “dementia”, “Traditional Chinese Medicine”, “natural product”, “herbs”, “botanical drug”, and “interactions”. Publications from the inception of these databases up to 2025 were included. We specifically focus on recent research findings, particularly those from the past 5 years, to ensure that the cited studies are contemporary and forward-looking. with an emphasis on recent studies. During the literature selection process, we first conducted an initial screening of the search results by reviewing the titles and abstracts to exclude articles that were clearly irrelevant or of low quality. For the studies that met the preliminary selection criteria, we performed a detailed full-text review to ensure that the included research could provide valuable insights into the topic of this review.

## 3 Communication between the gut microbiota and the brain

**FIGURE 1:**
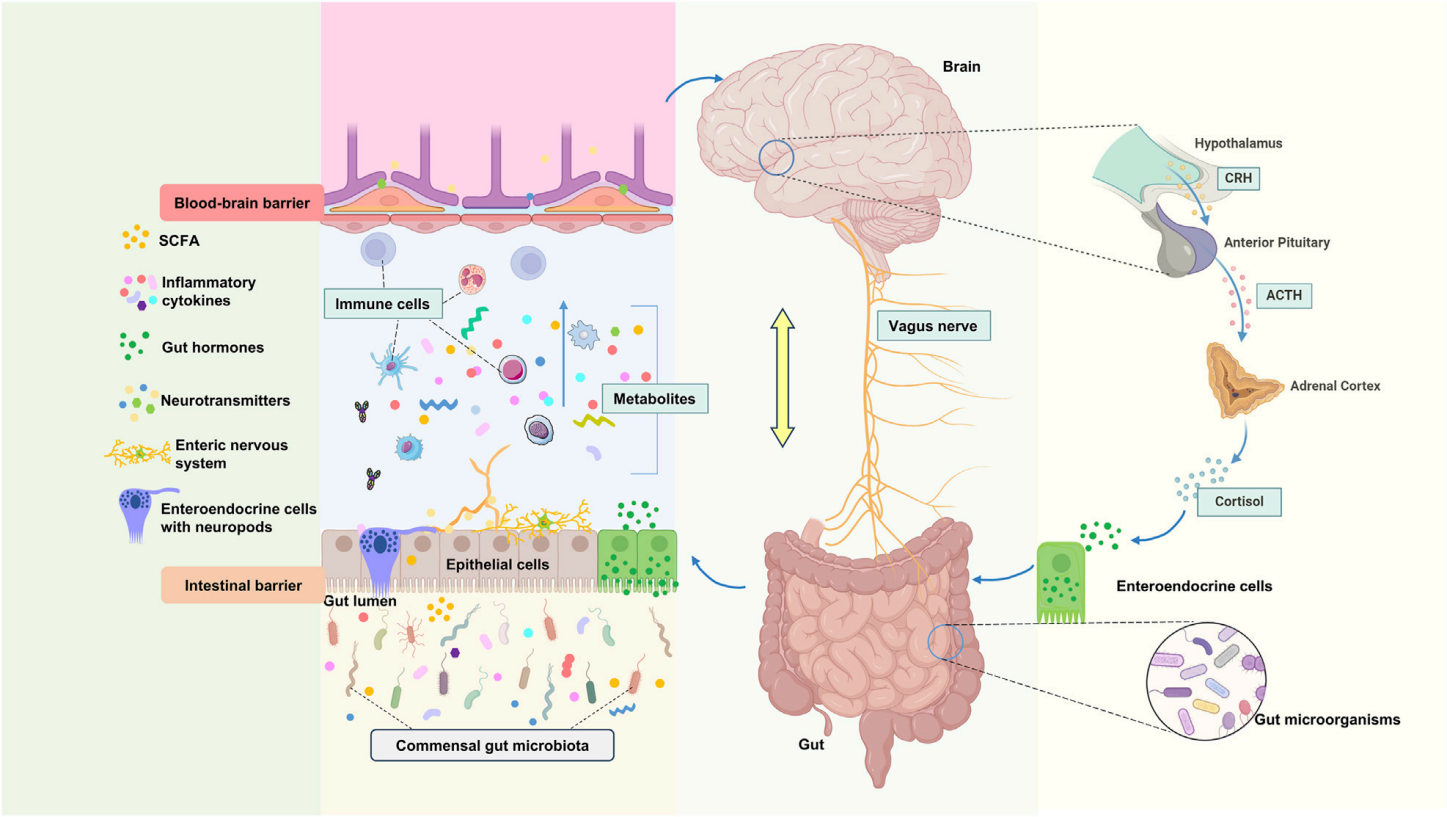
Physiological connection of microbial-gut-brain axis. CRH: Corticotropin-Releasing Hormone, ACTH: Adrenocorticotropic hormone.

The bidirectional communication between gut microbiota and the brain is mediated by the gut-brain axis (GBA), a multifunctional network involving the central, peripheral, immune, and endocrine systems. This axis maintains homeostasis among the gastrointestinal, neural, and microbial systems through various pathways, including chemical and neural transmission (Hashimoto, 2023). The brain can influence the microbiota indirectly via gut motility, metabolites, and intestinal permeability, or directly by releasing signaling molecules from innate cells into the intestinal lumen. Conversely, the microbiota affects brain function by synthesizing or releasing neurotransmitters, neuromodulators, and other metabolites. This interaction is closely linked not only to neural transmission but also to metabolic products, endocrine regulation, and immune modulation (Figure 1).

### 3.1 Vagus nerve as a direct link between gut microbiota and the brain

The vagus nerve (VN) is a mixed nerve that facilitates communication between the brain and various organs, comprising approximately 80% afferent fibers and 20% efferent fibers (Goggins et al., 2022). Originating from the medulla oblongata, it extends through the neck, chest, and abdomen, innervating most internal organs. Anatomically, the VN directly connects the central nervous system (CNS) with the gastrointestinal tract, housing multiple receptors sensitive to mechanical, chemical, and hormonal stimuli from gut microbiota (Liu et al., 2020). Thus, the VN can detect inflammatory chemicals, dietary components, microbial metabolites, and regulatory gut peptides through its receptors and afferent fibers (Martin et al., 2018; Ullah et al., 2023), transmitting gut information to the dorsal motor nucleus via the nucleus of the solitary tract. These signals are processed by the central autonomic network and projected to relevant cortical areas, ultimately forming cholinergic synapses with enteric neurons to respond to gut signals, regulating motility, secretion, and anti-inflammatory reflexes, thereby governing the gastrointestinal and enteric nervous systems (ENS) (Troadec et al., 2022). Consequently, the VN serves as a critical pathway for communication between gut microbiota and the brain (Figure 1).

Early studies have confirmed the significant role of the VN in gastrointestinal neural signaling. Bravo JA demonstrated that regulatory signals from *Lactobacillus rhamnosus (JB-1)* are processed via the VN and projected to the hippocampal region, enhancing GABA receptor expression in the hippocampus and improving anxiety- and depression-related behaviors (Bravo et al., 2011). Although the involvement of other neurotransmitter and neuropeptide systems cannot be excluded, it is undeniable that the VN is a key link between the brain and microbiota. A recent study (Yun et al., 2020) found that the probiotic *Lactobacillus gasseri* mediates gut-brain signaling through the VN, alleviating cognitive impairment and depression in mice while improving *Escherichia coli* (*E. coli*)-induced gut dysbiosis and inhibiting IL-1β expression. Additionally, other probiotics such as *Bifidobacterium longum NCC3001* (Bercik et al., 2011), *Lactobacillus reuteri* (Sgritta et al., 2019), and *Lactobacillus intestinalis* (Wang S. et al., 2020) also engage the VN pathway in gut-brain communication. Short-chain fatty acids (SCFAs) synthesized by the gut microbiota also inhibit the brain‘s feeding through the VN (Goswami et al., 2018).

### 3.2 Neurotransmitters and metabolites as material foundations for gut-brain communication

Gut microbiota interact with intestinal epithelial enteroendocrine cells to produce various neuroactive compounds and stimulate the host to synthesize additional metabolites and neurotransmitters, thereby regulating gut-brain signaling (Chen M. et al., 2022). These substances primarily include SCFAs, gamma-aminobutyric acid, norepinephrine, serotonin, glutamate, dopamine, and histamine, all of which act as chemical messengers in the brain that modulate neuronal activity through neurotransmitter synthesis or precursor levels (Angelucci et al., 2019; Rusch et al., 2023; Zou et al., 2023). They can also initiate ascending signaling pathways by diffusing through ENS nerve fibers, thereby mediating gut-brain communication and regulating neural, immune, and endocrine systems (Jia X. et al., 2023; Mitra et al., 2023). Microorganisms such as *yeasts*, *streptococci*, *bacilli*, *lactobacilli*, *E. coli*, and *bifidobacteria* are known to produce these metabolites or neurotransmitters (Han et al., 2022).

SCFAs are among the most extensively studied metabolites. Their primary components, propionate and butyrate, can regulate the levels of norepinephrine, epinephrine, the dopamine biosynthetic enzyme tyrosine hydroxylase (TH), and the serotonin synthetic enzyme tryptophan hydroxylase 1 (TPH1). Moreover, SCFAs stimulate enteroendocrine cells to release various neuropeptides and hormones, including cholecystokinin (CCK), glucagon-like peptide-1 (GLP-1), and leptin. These signaling molecules interact with the vagus nerve, thereby influencing the gut-brain axis. Additionally, SCFAs significantly influence central nervous system development and microglial activity (Deng et al., 2024). Microbial metabolites also regulate the levels of nerve growth factor (NGF), brain-derived neurotrophic factor (BDNF), and glial cell line-derived neurotrophic factor (GDNF), vital for neuronal and synaptic growth, survival, and differentiation. These metabolites also modulate cognitive function, memory, mood, and social behavior. In summary, neurotransmitters and metabolites produced by gut microbiota serve as the material basis for gut-brain communication, contributing to the regulation of gut-brain axis balance, control of immunity, modulation of neuronal activity, protection of intestinal mucosal barriers, and maintenance of endocrine homeostasis (Zhao H. et al., 2022).

### 3.3 Immune regulation as a key mechanism for gut microbiota and brain homeostasis

The relationship between microbiota and the host immune system begins early in life, with microbial contact and interaction promoting the development of the host immune system (Abdel-Haq et al., 2019; Donald and Finlay, 2023). After maturation, the release of specific antibodies and pro-inflammatory mediators involved in regulating brain immunity also relies on the interaction between the microbiota and the host immune system (Liu et al., 2020). In the CNS’s immune processes, gut microbiota can affect immune cell maturation and functionality, such as phagocytosis, antigen presentation, cytokine production, and inflammatory activation, ultimately affecting neurophysiological processes, including BBB stability and neurotransmitter synthesis (Fung et al., 2017; Liu et al., 2020).

Beyond the CNS, peripheral immune pathways are also considered important mechanisms through which microbiota regulate brain function and behavior. Metabolites originating from gut microbiota (such as tryptophan, indole, and SCFAs) have regulatory effects on gut immune cells and mucosal immunity, influencing peripheral immune responses and impacting brain inflammation and behavior (Hu T. et al., 2020; Ruohtula et al., 2019). The BBB is vital for regulating the balance between the peripheral circulation and the CNS. Dysbiosis can compromise its physical and chemical barrier functions, allowing immune cells and pro-inflammatory mediators from the bloodstream to penetrate the BBB and affect the CNS (Morais et al., 2021). Critically, the gut microbiome influences neuroinflammation through dual routes: (1) by modulating the function of resident CNS immune cells (e.g., microglial phagocytosis, cytokine production, and astrocytic antigen presentation), and (2) by promoting the trafficking of peripheral immune cells (such as monocytes and T cells) into the brain parenchyma via BBB disruption.

### 3.4 The hypothalamic-pituitary-adrenal (HPA) axis as a mediator in gut-brain feedback signaling

The HPA axis plays a central role in mediating stress responses and neuroendocrine activities within the gut-brain interaction, significantly impacting gut function and microbial composition. Simultaneously, the gut microbiota can influence the HPA axis through various mechanisms, facilitating bidirectional communication between the gut and brain (Chakrabarti et al., 2022; Mitra et al., 2023). The HPA axis responds to diverse stressors (e.g., emotional or physical stress), leading to adrenocorticotropic hormone (ACTH) secretion by the pituitary, which subsequently prompts the adrenal glands to produce cortisol. Cortisol subsequently increases the release of catecholamine neurotransmitters in multiple brain regions, enhancing noradrenergic activity (Kasarello et al., 2023; Misiak et al., 2020). These signaling molecules influence gut microbiota composition via several pathways. For instance, an animal study demonstrated that ACTH significantly affected the presence of *Ruminococcus*, *Klebsiella*, *Akkermansi*a, and *Lactobacillus* in the gut microbiome of rats (Song et al., 2019). Additionally, exposure to stressors was shown to affect the relative abundance of bacterial families such as Porphyromonadaceae, Lactobacillaceae, Ruminococcaceae, Coriobacteriaceae, Streptococcaceae, and Enterobacteriaceae (Delaroque et al., 2021; Sun Y. et al., 2013).

Furthermore, the gut microbiota can modulate the HPA axis by increasing gut barrier permeability or inducing pro-inflammatory states. Pro-inflammatory cytokines (e.g., IL-1, IL-6, and TNF-α) and bacterial metabolites (e.g., lipopolysaccharides [LPS], SCFAs, and peptidoglycans) can activate the HPA axis (Farzi et al., 2018). Moreover, gut microbiota modulates the levels of BDNF, corticotropin-releasing factor (CRF), NMDAR2 subtypes, and 5-HT1a receptors in the hippocampus and hypothalamus, which lead to functional shifts in the HPA axis (Mitra et al., 2023; Tooley, 2020). Specifically, microbial metabolites like short-chain fatty acids (SCFAs) directly regulate HPA activity by suppressing hypothalamic CRF release via epigenetic mechanisms (e.g., HDAC inhibition), thereby dampening stress responses (Kasarello et al., 2023; O'Riordan et al., 2022). Simultaneously, vagal afferent neurons detect gut-derived signals (including SCFAs) and relay them to the nucleus tractus solitarius (NTS), which projects to CRF-producing neurons in the hypothalamic paraventricular nucleus (PVN), further modulating HPA output (Cao et al., 2024; Herman, 2018). Dysregulation of the HPA axis can result in abnormal cortisol secretion, leading to hyper- or hypo-responsiveness to stress and disrupting circadian rhythms (de Weerth, 2017). HPA axis dysfunction is associated with cognitive decline, aging, immune system dysregulation, and systemic inflammation. Notably, cortisol dysregulation is strongly linked to the onset of neuropsychiatric symptoms (Misiak et al., 2020; Rusch et al., 2023). Additionally, gut microbes can modulate intestinal steroidogenesis, alter stress hormone levels, and transmit hormonal signals to the brain through extrarenal steroidogenesis, the autonomic nervous system, and various bacterial components. This allows brain regions to respond appropriately and regulate host physiological functions (Lai et al., 2023).

## 4 Gut microbiota and AD

**FIGURE 2:**
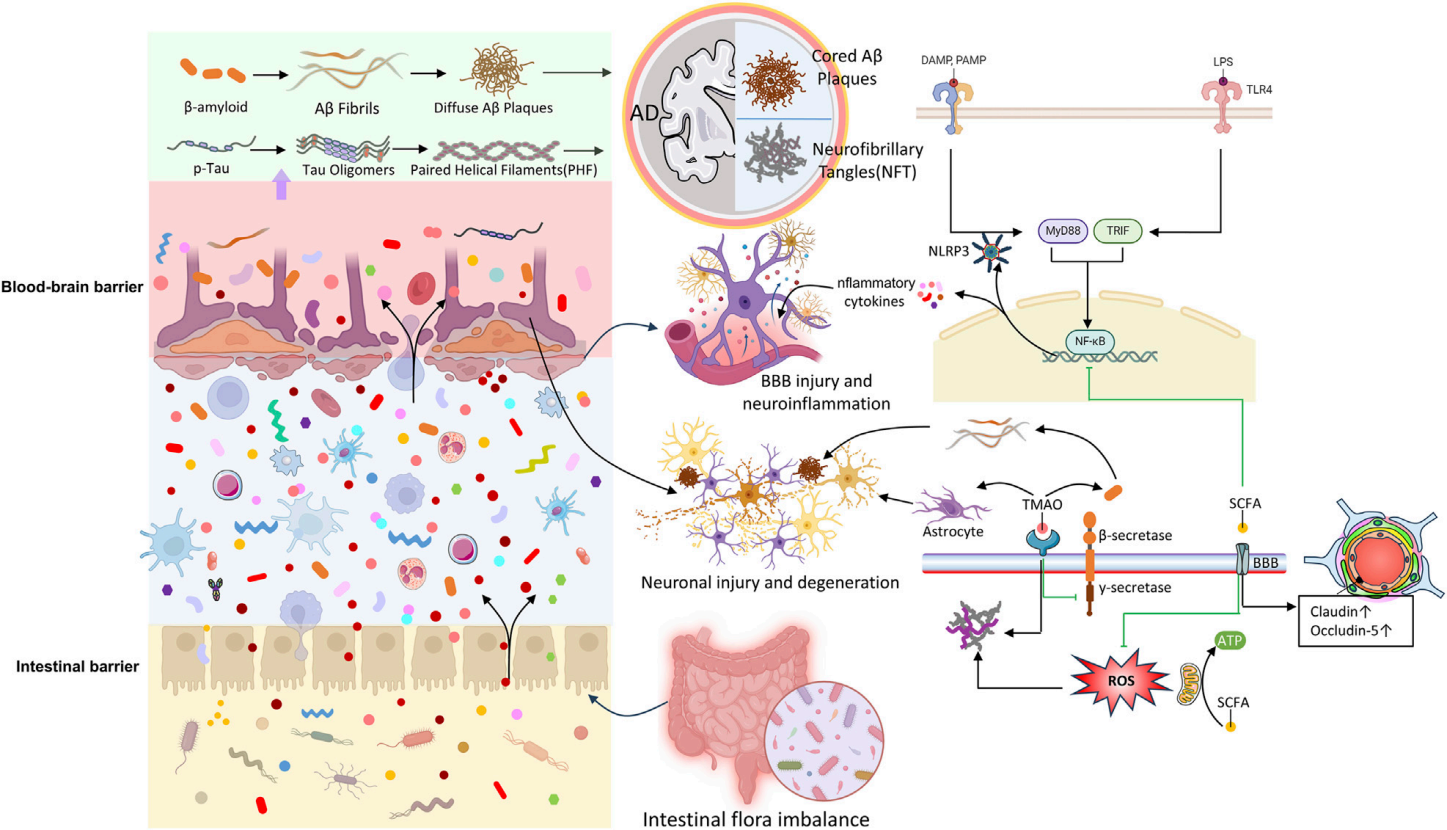
The pathological relationship between microbiota-gut-brain axis and AD.

While the interplay of genetic and environmental factors complicates the understanding of AD pathogenesis, increasing evidence shows that gut microbiota is central to various biological pathways influencing the onset and progression of AD. There is a notable link between gut microbiota and the two hallmark pathological features of AD: amyloid-beta (Aβ) and tau protein. These mechanisms involve synaptic plasticity, neuronal function, inflammation, oxidative stress, and barrier integrity. Recent clinical and experimental research has demonstrated the impact of gut microbiota on AD (Qu et al., 2024; Xiao et al., 2021). Interventions such as germ-free animal models, fecal microbiota transplantation (FMT), antibiotics, and probiotics have revealed the gut microbiota’s contribution to host cognition and AD-related pathophysiology. Although previous sections have explored the connection between gut microbiota and the brain, our understanding of the specific mechanisms in the context of AD remains limited. This section summarizes potential associations between gut microbiota and AD (Figure 2), with particular emphasis on how gut microbiota influences AD development.

### 4.1 Bacterial amyloid proteins

Microbial amyloid proteins, including amyloid peptides and curli-type amyloid fibrils, can promote the aggregation of neuronal amyloid proteins through cross-seeding. These proteins also activate inflammatory pathways involved in AD pathogenesis, accelerating disease progression (Miller et al., 2020; Shabbir et al., 2021). Among gut microbiota, *Escherichia coli* is a major contributor to amyloid production, with its amyloid proteins sharing structural and immunogenic similarities to Aβ42. Other bacteria, such as *Pseudomonas fluorescens*, *Staphylococcus aureus*, *Salmonella typhimurium*, *Bacillus subtilis*, and *Mycobacterium*, also produce amyloid proteins like curli, CsgA, FapC, TasA, and phenol-soluble modulins. Although these proteins differ in sequence from Aβ, they can trigger pathogenic mechanisms related to AD, promoting amyloid plaque deposition and facilitating Aβ fibril and oligomer misfolding. Misfolded proteins can then travel to the brain through the GBA (Elkins et al., 2024; Kim et al., 2019; Shabbir et al., 2021; Uceda et al., 2023).

In addition, bacterial amyloids interacting with the intestinal mucosa can provoke immune responses, compromising gut barrier integrity and promoting systemic inflammation. These responses exacerbate CNS inflammation by activating glial cells, stimulate the endogenous production of neuronal amyloid proteins (Megur et al., 2020; Tarawneh and Penhos, 2022; Toledo et al., 2022). Bacterial amyloids, when recognized by innate immune receptors, can trigger the upregulation of pro-inflammatory cytokines (e.g., TNF-α, IL-6, IL-1β, and nitric oxide), primarily via pathways involving TLR2/1, CD14, NF-κB, and iNOS (Tursi and Tükel, 2018). Additionally, bacterial amyloids can activate the NLRP3 inflammasome through Toll-like receptor 2 (TLR2), leading to the release of downstream pro-inflammatory factors by microglia, which ultimately contribute to Aβ and tau aggregation (Ising et al., 2019; Tejera et al., 2019; Venegas et al., 2017). Increased intestinal permeability exacerbates this process by allowing bacterial metabolites, including immunogenic amyloids and LPS, to enter systemic circulation. These substances promote neuroinflammation in the brain by increasing iNOS and NF-κB levels (Carloni and Rescigno, 2022; Pluta et al., 2020; Zhang Y. et al., 2021). In summary, gut dysbiosis contributes to both the production and accumulation of amyloid proteins and the neuroinflammatory processes associated with AD (Ağagündüz et al., 2022; Pistollato et al., 2016).

### 4.2 Tau hyperphosphorylation

Hyperphosphorylation of tau, resulting in neurofibrillary tangles (NFTs), is a key feature of AD and is strongly associated with cognitive deterioration, in parallel with Aβ pathology. Trimethylamine N-oxide (TMAO), a metabolite derived from gut microbiota, has been shown to correlate with tau pathology, with increased TMAO concentrations observed in the cerebrospinal fluid (CSF) of AD patients, and a positive relationship with NFTs (Vogt et al., 2018). Additionally, studies have also indicated an increased abundance of Blautia and *Bacteroides* in AD patients, which correlates positively with CSF p-tau concentrations, whereas SMB53 and Dialister exhibit a notable decline and are inversely related to CSF p-tau levels (Vogt et al., 2017). In tauopathy mouse models, reduced abundances of *Lactobacillus*, *Streptococcus*, *Marvinbryantia*, and the *Eubacterium brachy* group were negatively associated with tau pathology in the brain, suggesting that alterations in these microbial populations may be closely linked to tau-related neurodegeneration (Sun et al., 2019).

*Helicobacter pylori* has also been implicated in tau hyperphosphorylation, as it induces tau phosphorylation through the activation of glycogen synthase kinase-3β (GSK-3β) (Kim et al., 2021). Extracellular vesicles from gut bacteria, which are integral to modulating the intestinal environment, have been shown to provoke inflammation and tau hyperphosphorylation via GSK-3β pathway activation, contributing to cognitive decline (Liang et al., 2022). In tauopathy animal models, reductions in the relative abundances of *Bacteroides*, *Lactobacillus*, and *Alistipes* have been observed. Notably, antibiotic treatment or FMT has been demonstrated to modify the gut microbiota and reduce pathological tau in these models (Zhang et al., 2023c; Zhu Y. et al., 2024).

### 4.3 Neuroinflammation

Neuroinflammation is a protective response of the CNS (Chen et al., 2024b). However, in AD, chronic and sustained inflammation surpasses physiological limits, significantly impairing Aβ clearance and compromising BBB permeability. This exacerbates neuronal and glial apoptosis, synaptic dysfunction, and the accumulation of toxic substances such as Aβ and tau proteins (Jemimah et al., 2023; Ghezzi et al., 2022; Saji et al., 2020). These outcomes perpetuate a vicious cycle of neuroinflammation, with gut microbiota emerging as a potential key driver (Chidambaram et al., 2022). Several studies (Alsegiani and Shah, 2022; Jemimah et al., 2023; Zhou et al., 2022) have demonstrated that gut dysbiosis promotes neuroinflammation by activating inflammasome signaling pathways. Microglia, as critical mediators, respond to Aβ deposition by recognizing damage-associated molecular patterns (DAMPs) and pathogen-associated molecular patterns (PAMPs) via surface receptors, triggering inflammatory cascades that release pro-inflammatory mediators and toxic by-products, including nitric oxide (NO), reactive oxygen species (ROS), and cytokines (Margolis, 2015; Pena-Ortega, 2017). This results in gut barrier disruption, neuroinflammation, synaptic dysfunction, and neuronal loss (Jin et al., 2023). As previously discussed, bacterial amyloid proteins play a pivotal role in this process.

Furthermore, alterations in gut microbiota and its metabolites can modify gene and protein expression, promoting the accumulation of inflammatory proteins in the brain and inducing neuroinflammation (Alsegiani and Shah, 2022). For instance, genes encoding innate immune proteins, such as CD33, TREM2, and CR1, have been shown to reduce microglial phagocytic activity and impair Aβ clearance when altered, as confirmed by genome-wide association studies (GWAS) as key elements in AD-related inflammatory pathology (De Marchi et al., 2023; McQuade and Blurton-Jones, 2019; Pampuscenko et al., 2023). In addition to microglia, astrocytes are also key participants in neuroinflammation. Continuous Aβ deposition stimulates astrocytes to release cytokines, further compromising BBB integrity and amplifying the inflammatory cascade, ultimately promoting neurodegeneration (Rothhammer and Quintana, 2015). In addition to microglia, astrocytes are also key participants in neuroinflammation. Continuous Aβ deposition stimulates astrocytes to release cytokines, further compromising BBB integrity and amplifying the inflammatory cascade, ultimately promoting neurodegeneration (Rothhammer and Quintana, 2015). Critically, astrocytes and microglia engage in bidirectional crosstalk: microglial priming by DAMPs/PAMPs (e.g., bacterial LPS or Aβ) triggers TNF-α, IL-1α, and C1q release, which induces reactive astrogliosis and inflammatory cytokine production in astrocytes. In turn, reactive astrocytes amplify microglial activation via CCL2 and complement component C3, creating a feed-forward loop that sustains neuroinflammation and impairs Aβ clearance (Han et al., 2025; Mishra et al., 2021; Rao et al., 2025).

Lipopolysaccharide (LPS)-induced inflammatory pathways also play a significant role in the interaction between gut microbiota and AD. LPS, primarily generated by Gram-negative bacteria, can breach the intestinal barrier during dysbiosis, enter systemic circulation, and stimulate immune cells like macrophages and dendritic cells. By binding to immune cell receptors (TLR2, TLR4, and/or CD14), LPS activates MyD88-and NF-κB-dependent signaling pathways, leading to a cytokine and chemokine cascade that triggers neuroinflammation (Biscari et al., 2022; Lai et al., 2021; Lukiw, 2016). Additionally, LPS can directly affect neurons, increasing excitability and disrupting synaptic transmission, thereby intensifying neuroinflammation. It also promotes neuronal damage by inducing apoptosis and synaptic injury, accelerating AD progression. Recent research has established a causal relationship between LPS-induced neuroinflammation, Aβ aggregation, and memory impairment. Moreover, LPS has been shown to activate astrocytes and reduce BDNF expression (Wu et al., 2021).

### 4.4 Barrier disruption

Damage to the gut and blood-brain barriers, often driven by gut microbiota dysregulation and abnormal metabolite production, is a critical factor in AD-related neuroinflammation. The relationship between barrier dysfunction and neuroinflammation is bidirectional. Compromise of the intestinal epithelial barrier allows pathogenic microbes to translocate unchecked, introducing inflammatory mediators into peripheral circulation and facilitating their dissemination into the CNS through a compromised BBB (Pellegrini et al., 2018). It is reported that increased peripheral levels of IL-1β and TNF-α have been positively associated with AD risk (Davinelli et al., 2011). Clinical research by Annamaria Cattaneo et al. found that elevated plasma levels of NLRP3, IL-1β, and CXCL2 in patients with cognitive impairment and cerebral amyloidosis were strongly linked to higher levels of pro-inflammatory bacteria (*Escherichia coli*/*Shigella*) and reduced levels of anti-inflammatory bacteria (*Faecalibacterium rectale*) (Cattaneo et al., 2017). This perspective was further supported by Divya Goyal et al. (Goyal et al., 2021). Moreover, structural and functional disruption of the BBB has been observed early in AD progression (Morris et al., 2023; Steinman et al., 2020), and research suggests that restoring gut microbial homeostasis can restore BBB integrity. This highlights the interconnected nature of peripheral inflammation and AD through barrier disruption, with gut microbiota playing a central role in this process.

### 4.5 Oxidative stress

Oxidative stress results from a disruption in the balance between ROS production and clearance. The gut microbiota significantly influences redox homeostasis by synthesizing metabolites, regulating antioxidant enzymes, and maintaining gut equilibrium. Gasotransmitters such as NO, hydrogen sulfide (H_2_S), and hydrogen (H_2_) are key regulators of redox balance and gut microbiota, playing significant roles in AD pathogenesis. *Lactobacillus*, *Escherichia coli*, and *Bifidobacterium* convert nitrates and nitrites into NO, which reacts with superoxide to generate peroxynitrite, a potent oxidant that contributes to AD-related neurotoxicity (Das and Ganesh, 2023). Pathogenic bacteria like *Salmonella* and *E. coli* break down sulfur-containing amino acids to produce H_2_S. At high concentrations, H_2_S inhibits cyclooxygenase activity, reduces mitochondrial oxygen consumption, and exacerbates pro-inflammatory effects (Beaumont et al., 2016). Hydrogen, primarily produced by anaerobic cocci, *Clostridium* species, and members of the Enterobacteriaceae family, has antioxidant properties. Gut dysbiosis may reduce hydrogen production, limiting its availability to neurons in the CNS. Additionally, SCFAs, such as butyrate, possess strong antioxidant properties, helping to eliminate ROS and enhance the host’s antioxidant defenses (Munteanu et al., 2024). Dysbiosis can disrupt redox balance, accelerating AD-related pathological processes. Notably, pro-inflammatory cytokines and kynurenine pathway metabolites can induce ROS bursts, leading to additional damage to glial cells and aggravating neuronal injury, thus establishing a harmful cycle that accelerates neurodegeneration (Wu et al., 2021). In summary, gut microbiota influences oxidative stress, impacting host immunity and inflammation, and plays a significant role in the pathogenesis of AD.

## 5 Traditional use of TCM(Plants) in treating AD

TCM, with its distinct theoretical framework, emphasizes a holistic approach to treatment by restoring the body’s yin-yang balance through dialectical methods. One of the primary therapeutic modalities in TCM is the oral administration of herbal decoctions, whose efficacy has been validated for thousands of years. To this day, TCM continues to yield remarkable results in treating complex diseases, although the precise mechanisms behind these effects remain incompletely understood by modern scientific and medical techniques. This, perhaps, represents the allure of TCM, which also presents both opportunities and challenges for researchers.

TCM has long utilized herbal formulations and plant-derived metabolites to address cognitive decline and neurological disorders, including symptoms now recognized as AD. In TCM, AD is classified under the category of “dementia,” primarily attributed to kidney essence deficiency and the accumulation of phlegm and blood stasis, while also being linked to emotional and environmental factors. Consequently, treatment strategies often focus on “tonifying the kidney and enriching essence,” “activating blood and removing stasis,” “regulating qi and resolving phlegm,” and “strengthening the spleen and nourishing the heart.” Among these, “regulating qi and resolving phlegm” is a therapeutic strategy targeting two interconnected pathological elements: Regulating qi restores the flow and functional activity of qi (vital energy), resolving stagnation that disrupts organ function (e.g., digestion, pain regulation); Resolving phlegm eliminates pathological accumulations of bodily fluids, which manifest as substantial phlegm (e.g., sputum) or non-substantial phlegm (e.g., cognitive fog, metabolic disorders). Rooted in the holistic principles of “the unity of heaven and man” and “multi-target treatment,” TCM emphasizes the interaction between the body, mind, and environment, using the synergistic effects of herbal combinations to address underlying systemic imbalances and restore physiological equilibrium.

Several medicinal plants in TCM have been used for centuries to treat conditions resembling those of AD, driven by the belief that these plants possess the ability to nourish the brain, invigorate blood circulation, and eliminate toxins that may accumulate in the brain. For example, *Ginkgo biloba*, one of the most well-known plants in both Western and Eastern herbal medicine, has been traditionally used to improve memory and enhance cognitive function. Its leaves contain flavonoids and terpenoids, which are thought to improve blood circulation and protect against neuronal damage caused by oxidative stress (Zhu C. et al., 2024), a key mechanism in AD pathology. Another example is *Panax ginseng*, long known for its cognitive-enhancing properties. Studies (Lee et al., 2024) indicate that ginsenosides in ginseng may reduce amyloid-β aggregation, inhibit neuroinflammation, and protect neuronal integrity—pathological features commonly observed in AD. Similarly, *Reynoutria multiflora*, known for its “tonifying the liver and kidney” and “nourishing the blood” effects, has been frequently included in prescriptions. Some studies suggest that its active metabolites, such as resveratrol, may modulate oxidative stress and inflammation in the brain, potentially alleviating cognitive decline associated with AD (Cha et al., 2024; Liu et al., 2025; Surya et al., 2025). *Carthamus tinctorius*, another botanical drug traditionally used in TCM to promote blood circulation and alleviate cognitive impairment, has recent studies (Liang and Wang, 2022) showing that its flavonoid metabolites may exert neuroprotective effects by reducing neuroinflammation and promoting neuronal survival—mechanisms highly relevant to AD treatment. *Epimedium sagittatum*, employed for its tonic effects in TCM, is believed to enhance blood circulation and support cognitive function in AD by improving cerebral blood flow, thereby helping to restore impaired brain function. Furthermore, gastrodin, derived from *G. elata* (Tianma), has been used for centuries to “calm wind and unblock collaterals,” reflecting its historical role in alleviating tremors and cognitive impairment. Finally, *Rheum palmatum*, traditionally used to clear heat, detoxify, and promote bowel movements, has recent research (Gao et al., 2021) suggesting its role in amyloid plaque clearance and reduction of neuroinflammation, processes associated with AD pathology.

In addition to these individual plants, several classic TCM herbal formulas have traditionally been used to treat cognitive disorders similar to AD. For instance, Qi Fu Yin is a well-known formula used to tonify the heart and spleen, often associated with age-related cognitive decline. This formula aims to invigorate blood and nourish the brain, with key botanical drugs such as *Rehmannia glutinosa* and *Angelica sinensis* believed to have neuroprotective effects (Ong et al., 2018). Kai Xin San is another traditional formula commonly used in TCM to alleviate symptoms such as forgetfulness and mental fatigue. This formula, composed of botanical drugs like *P. ginseng* and *Polygala tenuifolia*, has been shown to improve memory, reduce anxiety, and enhance cognitive function, potentially benefiting AD patients (Chen L. et al., 2024). Another important formula is Liu Wei Di Huang Wan, traditionally used to tonify the kidney and liver, organs crucial for cognitive health in TCM. It contains *R. glutinosa*, *Cornus officinalis*, and other botanical drugs believed to promote blood circulation and alleviate neurodegeneration, benefiting brain health (Wang et al., 2017). Additionally, Banxia Xiexin Decoction, a well-known formula recorded in the “Shang Han Lun,” is traditionally used to treat “pi syndrome,” characterized by digestive disturbances—symptoms overlapping with neuroinflammation and gut dysbiosis seen in AD (Liao et al., 2023). Modern studies suggest that its components, such as *Scutellaria baicalensis* (rich in baicalin) and *Coptis chinensis* (containing berberine), can regulate gut microbiome diversity, inhibit amyloid-β aggregation (Gao et al., 2024), and suppress neuroinflammatory pathways. Similarly, Chai Hu Shu Gan San, historically used to treat “liver qi stagnation” associated with emotional and cognitive dysfunction, contains *Bupleurum chinense* (with saikosaponins) and *Citrus reticulata* (rich in polymethoxyflavones), which may enhance gut barrier integrity and reduce tau hyperphosphorylation through microbiome-mediated metabolic regulation.

The therapeutic potential of these traditional plants and herbal formulas lies not only in their long history of use but also in the unique bioactive compounds they contain. These compounds, when absorbed and metabolized by the gut microbiota, may be further biotransformed into metabolites that enhance their therapeutic efficacy. By linking traditional herbal knowledge with modern microbiome science, it is possible to uncover new mechanisms by which these plants can be used to modulate gut-brain communication, restore balance to neuroinflammatory pathways, and promote cognitive health.

## 6 TCM and gut microbiota

As research interest in the gut microbiota grows, the study of TCM’s effects in this area has deepened. A key area of focus is how TCM may influence disease progression through mechanisms such as gut microbiota modulation, showing promising potential for the treatment of AD. The mechanisms through which TCM affects AD are multi-pathway and multi-target in nature. Whether through single botanical drugs, individual botanical drug components, herbal pairs, or complex herbal formulas, TCM can exert diverse cascading effects via a broad array of signaling pathways, including Wnt/GSK-3β/β-catenin, AMPK/Nrf2, BDNF-TrkB-ERK/Akt, NLRP3/caspase-1, RAGE/NOX4, and PI3K/Akt/GSK-3β. These actions contribute to reducing tau tangles, decreasing Aβ aggregation, inhibiting neuroinflammation, modulating oxidative stress, improving autophagy and apoptosis, alleviating synaptic dysfunction, and inhibiting acetylcholinesterase activity (Guan et al., 2022; Jin et al., 2019; Shan et al., 2024; Yang et al., 2022; Zhou et al., 2024).

Moreover, once orally administered, TCM interacts with the gut, where various factors influence its effects, including the chemical nature of herbal components, the activity of gut enzymes and transport proteins, the thickness and pH of the mucus layer, intestinal tissue structure, and the gut microbiota composition, influence its effects. These factors also include genetic differences and potential drug interactions. Furthermore, TCM influences the gut microbiota balance by optimizing its diversity and composition, which subsequently impacts brain activity via metabolites, neurotransmitters, and other bioactive substances. This connection is a central theme that will be further explored in the context of AD.

### 6.1 Influence of gut microbiota on the metabolism and absorption of TCM

**FIGURE 3:**
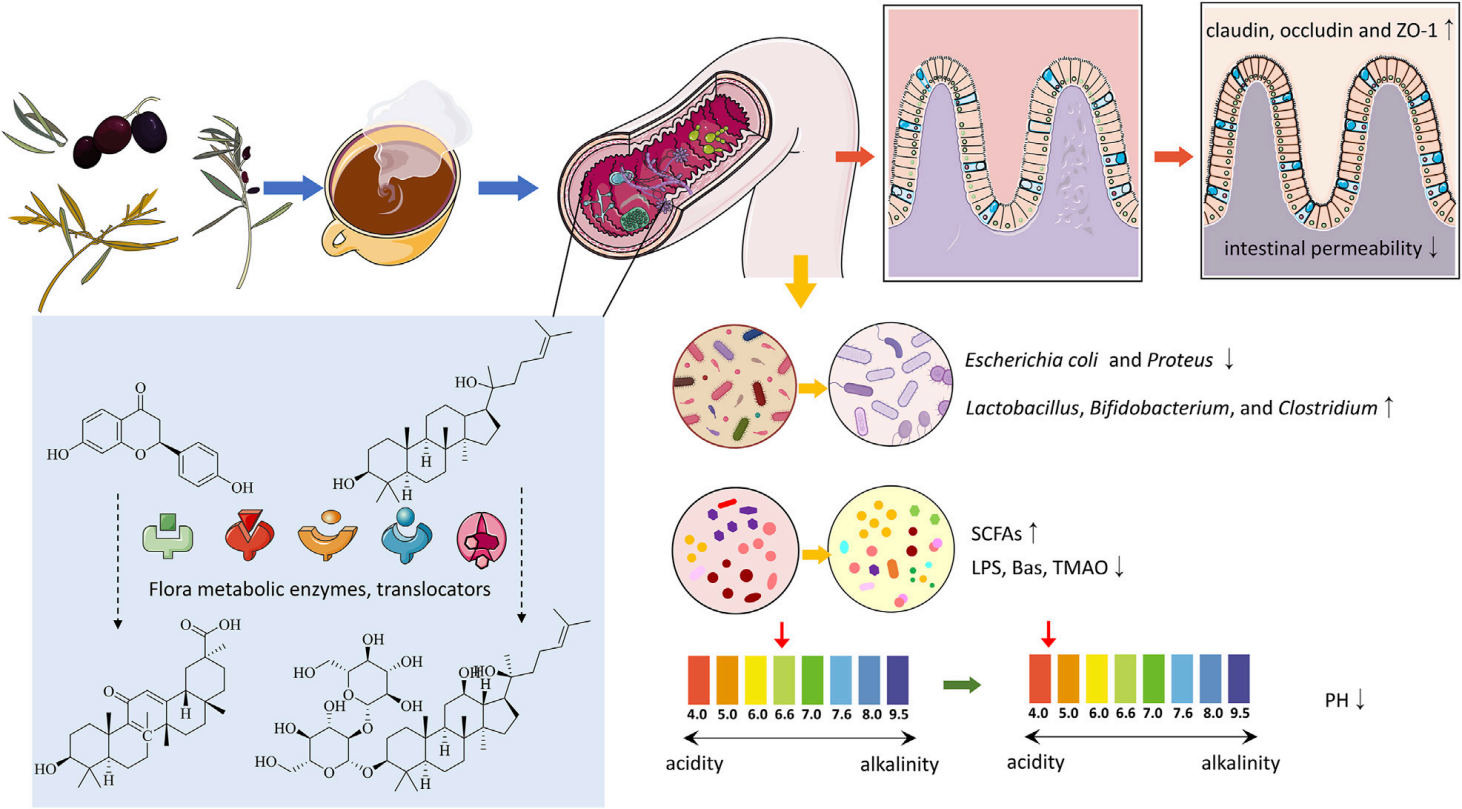
The interaction between traditional Chinese medicine and intestinal flora.

Studies show that gut microbiota can significantly affect the pharmacokinetics and pharmacodynamics of compounds in TCM (Weersma et al., 2020). The microbiota metabolizes TCM compounds through its enzymatic systems, while gut cells, via transport proteins and metabolic enzymes, further influence the absorption and metabolism of these compounds (Figure 3). The biotransformations carried out by the microbiota include hydrolysis, oxidation, reduction, and isomerization reactions, which alter the chemical structure, pharmacological activity, and toxicity of TCM compounds, generating new active metabolites. These metabolites, typically characterized by lower polarity and stable molecular weights, enhance the absorption and bioavailability of TCM, thereby increasing efficacy or reducing toxicity and producing diverse biological effects (Shen et al., 2013; Xu et al., 2017).

Specifically, gut microbiota affects the metabolism of TCM by influencing drug absorption, bioavailability, distribution, and clearance, with microbial enzymes playing a crucial role. For example, Zhao et al. demonstrated that the combined action of uridine diphosphate-glucuronosyltransferases (UGTs), efflux transporters, and gut microbiota enhanced the absorption and metabolism of galangin (Zhao F. et al., 2022). Additionally, metabolic enzymes expressed in the liver and gut, including cytochrome P450, sulfotransferases, and catechol-O-methyltransferases (COMTs), also play vital roles. Quercetin, for instance, has a relatively low oral absorption rate, but specific enzymes in the gut microbiota and intestinal epithelial cells metabolize quercetin and its derivatives into various metabolites, such as phenolic acids, which are subsequently absorbed, transformed, or excreted by the gut (Chen et al., 2023). An early study comparing the oral administration of liquiritigenin in germ-free and conventional rats showed that gut microbiota converts liquiritigenin into glycyrrhetinic acid for absorption. Recent *in vitro* studies have confirmed the essential role of gut microbiota in liquiritigenin metabolism and absorption (Takeda et al., 1996; Keranmu et al., 2022). Ginsenosides (Rb1, Rb2, and Rc), key metabolites of *P. ginseng*, are metabolized by gut microbiota into secondary metabolites (e.g., compound K, PPD, Rh2, and Rg3) after oral administration, exerting anti-inflammatory, antioxidant, and anti-apoptotic effects (Majnooni et al., 2023). Using RNA-seq, Feng et al. explored the regulatory effects of gut microbiota on the efficacy of Taohong Siwu Decoction (TSD) and identified a microbial metabolite that effectively inhibited DNA replication, highlighting the pivotal role of gut microbiota in determining the pharmacological outcomes of TCM (Feng et al., 2023).

### 6.2 The effects of TCM on gut microbiota

The active compounds in TCM interact with the gut microbiota directly or via metabolic conversion, selectively inhibiting or promoting the growth of specific microbial species to modulate the microbiota’s structure and function (Che et al., 2022). This interaction helps protect the intestinal mucosal barrier, maintain microbiota homeostasis, and improve brain function. The mechanisms by which TCM influences gut microbiota can be summarized into four primary aspects.

#### 6.2.1 Impact on the diversity and abundance of gut microbiota

The primary constituents of TCM, including alkaloids, phenolics, terpenoids, flavonoids, and organic acids, can inhibit the growth of pathogenic bacteria like *Escherichia coli* and *Proteus* while encouraging the growth of beneficial species like *Lactobacillus*, *Bifidobacterium*, and *Clostridium*, thereby altering the diversity and abundance of gut microbiota (Figure 3). For instance, the polysaccharides in Qiweibaizhu Powder have been reported to regulate the diversity, relative abundance, and community structure of the gut microbiota (Li C. et al., 2022). Through 16S sequencing, Qu et al. observed that atractylodin could modify the diversity and composition of the gut microbiota in colitis mice, and further microbial enrichment analysis suggested that its effects on the microbiota might be related to metabolic regulation (Qu L. et al., 2022). Phenolic metabolites in *Lycium chinense* have been found to enhance the diversity and richness of gut microbiota, promoting the growth of beneficial microbes such as *Bifidobacterium* and *Lactobacillus* while increasing the production of SCFAs that benefit gut health (Rajkowska et al., 2023; Zhou et al., 2017). Cistanche polysaccharides have similarly been shown to influence pharmacokinetic parameters and gut microbial composition (Fu et al., 2020). Recent research has demonstrated that Jianpi Tiaogan Decoction can influence the composition and functional properties of the gut microbiota in obese mice. Specific microbial changes, such as an increase in the Lachnospiraceae *NK4A136* group and *Oscillibacter*, were closely linked to therapeutic benefits (Dong et al., 2022).

#### 6.2.2 Influence on gut microbiota metabolism and function

Current research has established a significant relationship between TCM and the metabolic products of gut microbiota, including bile acids, SCFAs, amino acids, indoles, and their derivatives (Feng et al., 2018; Li J. et al., 2023; Wang K. et al., 2023). This highlights the importance of understanding how TCM supports gut microbiota stability and regulates microbial metabolites. TCM can influence the metabolism and functionality of gut microbiota, impacting nutrient absorption and energy metabolism in the human body. For instance, saponins from *Reynoutria japonica* are extensively fermented and metabolized by gut microbiota, promoting the growth of beneficial bacteria like *Bifidobacterium* and *Lactobacillus*, while suppressing harmful bacteria such as *Enterococcus* and *Escherichia coli*. This process enhances SCFA production, thereby improving the gut microbial environment (Figure 3) (Chai et al., 2021; Chen Z. et al., 2022). Other botanical drugs, including *Paeonia lactiflora* (Zhao et al., 2023), *R. glutinosa* (Lv et al., 2023), Lotus seeds (Dai et al., 2023), and *Taraxacum sinicum* (Yan et al., 2023), exhibit similar mechanisms that help rectify gut dysbiosis and promote SCFA production.

Additionally, formulations such as Gegen Qinlian Decoction have been shown to stimulate the production of propionate and total SCFAs, regulate medium- and long-chain fatty acids (M-LCFAs), maintain bile acid (BA) homeostasis, and modulate amino acid (AA) metabolism (Li Q. et al., 2021). A study investigating the effects of Shengjiang Xiexin Decoction on *Clostridium difficile* infection found that this formulation has the potential to regulate gut microbiota and balance bile acid metabolism (Yu et al., 2024). Furthermore, the lipid-lowering herbal formula Tangnaikang (TNK) has been demonstrated to modulate gut microbiota by upregulating *Akkermansia* and downregulating *Clostridium sensu stricto 1*, influencing fatty acid metabolism. Metagenomic analysis revealed that TNK is closely associated with fatty acid synthesis pathways (Tian et al., 2022). Liu et al. through integrated analysis of gut microbiota and liver lipid metabolomics, confirmed a correlation between microbial dysbiosis and altered lipid metabolites, suggesting that Buyang Huanwu Decoction may regulate hepatic lipid metabolism through gut microbiota while influencing gene and protein expression (Liu et al., 2022b).

#### 6.2.3 Impact on gut barrier and permeability

As previously noted, neuroinflammation is closely linked to the gut microenvironment and is a key pathological pathway leading to neurodegeneration. Under normal physiological conditions, gut bacteria and viruses are unable to cross the mucosal barrier. However, when gut dysbiosis or other factors induce intestinal inflammation, the permeability of the gut barrier increases, allowing more bacteria and viruses to translocate into the bloodstream. TCM active compounds can mitigate the effects of gut dysbiosis on the intestinal barrier by enhancing the gut microenvironment, including reducing inflammation and immune suppression, thereby preventing the progression of peripheral inflammation and maintaining CNS homeostasis (Figure 3). For instance, baicalin maintains gut immune homeostasis by protecting tight junctions (TJs) in the intestinal epithelium through the MLCK/pMLC2 signaling pathway (Huang et al., 2020). In addition, Jiawei Gegen Qinlian Decoction has been shown to protect the intestinal barrier by upregulating the expression of tight junction proteins (Li Q. et al., 2021). Similarly, a modified formula of Sishenwan, Fufangxiaopi, alleviated colitis-related symptoms in mice by regulating gut microbiota and significantly enhancing the expression of claudin-3, occludin, and ZO-1, thereby maintaining the intestinal barrier’s integrity (Liu K. et al., 2024).

Zhao et al. demonstrated that *Clostridium butyricum* helps restore the gut barrier by enhancing SCFA production following paeoniflorin treatment after antibiotic-induced gut microbiota depletion (Zhao et al., 2023). Duan et al. examined how *P. cocos* (Fuling) polysaccharides affect the intestinal mucosa in mice, finding that these polysaccharides regulate gut barrier function through microbial composition changes and increased expression of Wnt/β-catenin and Lrp5 proteins, thus supporting the physical, biochemical, and immune barriers (Duan et al., 2023). Additionally, berberine enhances glucagon-like peptide-2 (GLP-2) secretion, improves gut microbiota composition, increases mucin and antimicrobial peptide production, and upregulates the expression of mucin, occludin, and ZO-1 in insulin-resistant rats (Wang Y. et al., 2021).

#### 6.2.4 Influence on gut pH

Gut pH plays a vital role in shaping the microbiota by creating specific microenvironments favorable to various microbial species. The differing pH levels across the gastrointestinal tract form distinct niches that significantly influence the composition and abundance of the microbiota. TCM can modulate gut pH, thereby affecting microbial composition and function. Certain TCM compounds, such as polysaccharides, phenolic acids, and flavonoids, can alter gut pH (Figure 3), influencing the growth and metabolism of gut microbiota. For example, atractylodin from *Atractylodes lancea* can reduce gut pH, enhance the adhesion of Bifidobacterium to intestinal epithelial cells, and inhibit the proliferation of *E. coli* and *Enterococcus faecalis* (Shu et al., 2017). TCM can also influence gut pH by regulating the production of microbial metabolites, such as SCFAs, which lower pH and suppress the growth of pathogenic bacteria (Li C. et al., 2022).

For instance, ganoderma oligosaccharides promote the proliferation of acid-producing bacteria, thereby lowering gut pH (Xia et al., 2022). The *nelumbo nucifera* starch-lauric acid complex (LS12) has been shown to increase SCFA production through gut fermentation, reducing gut pH and creating an environment conducive to the growth of beneficial bacteria (Li D. et al., 2023). A study involving pigs demonstrated that a TCM formulation based on *Coix lacryma-jobi* and *Nelumbo nucifera* lowered gastrointestinal pH, increased villus height, promoted the growth of probiotics, and inhibited pathogenic microorganisms (Li Z. et al., 2021).

## 7 The influence of TCM on AD through interaction with gut microbiota

**FIGURE 4:**
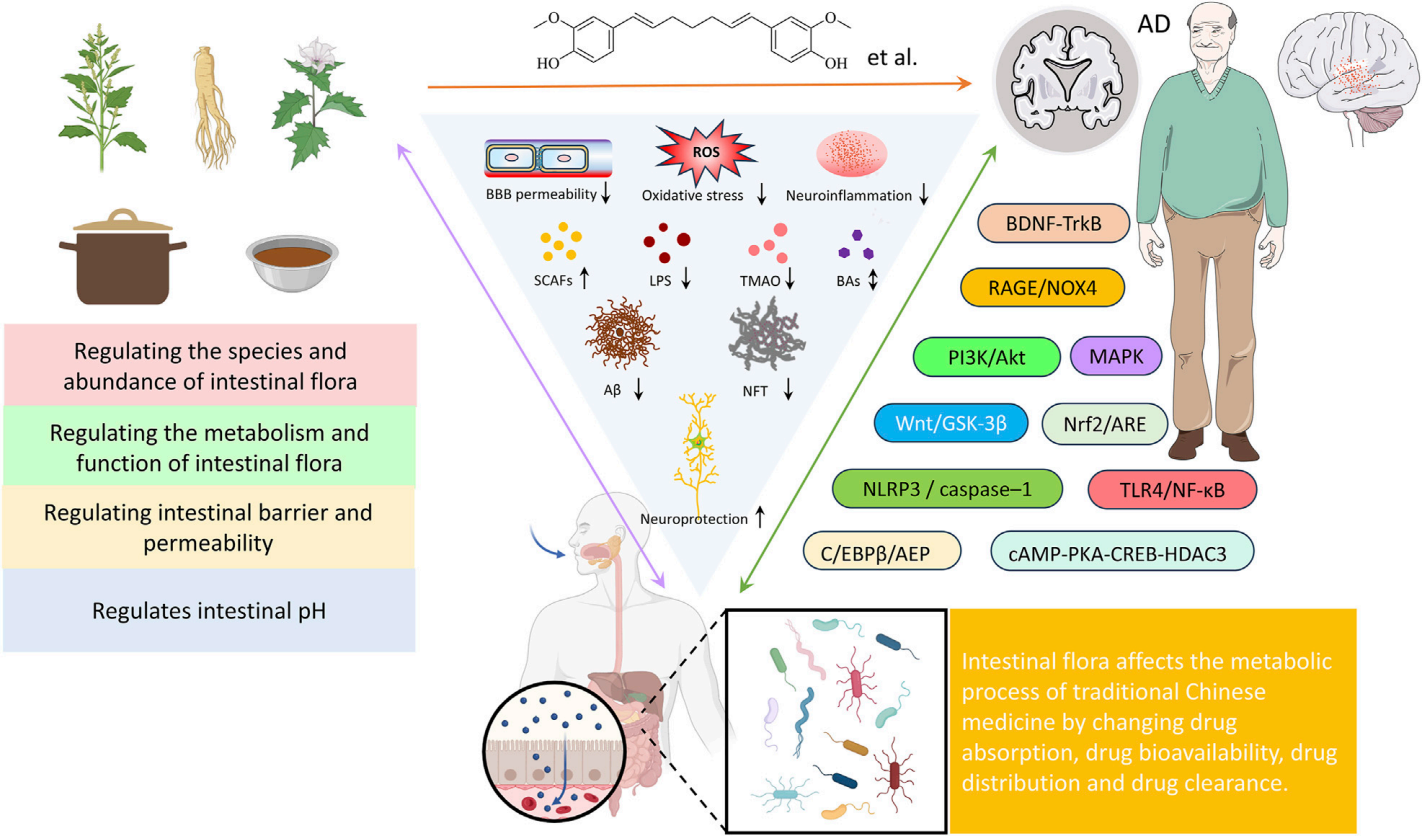
The influence of TCM on AD through interaction with gut microbiota.

TCM is a valuable component of Chinese medical heritage, and its potential in treating AD is increasingly attracting attention. While the specific mechanisms underlying TCM’s anti-AD effects remain incompletely understood, the gut microbiota and its interaction with the gut-brain axis is emerging as a key factor in TCM’s therapeutic approach to AD. Active compounds in Chinese botanical drugs can influence the gut microbiota, enhancing drug bioavailability and potentially generating multiple biological effects through modulation of microbiota structure and function (Figure 4). These effects include regulating neurotransmitter balance, improving behavioral and memory deficits, reducing amyloid-beta (Aβ) deposition, inhibiting acetylcholinesterase activity, alleviating neuronal apoptosis, and enhancing the brain’s antioxidant capacity (Figure 4). Thus, influencing the interaction between the gut-brain axis is regarded as a key mechanism underlying TCM’s effectiveness against AD. Whether used alone or as adjunctive therapy, TCM has demonstrated unique advantages in significantly improving cognitive function and quality of life in AD patients.

### 7.1 Regulation of gut microbiota

Studies have shown that numerous traditional Chinese medicines significantly influence the gut microbiota profile in AD (as summarized in Table 1). These effects are primarily characterized by an increase in beneficial bacteria (such as Firmicutes and Bifidobacterium) and a reduction in harmful bacteria (such as Bacteroidetes and *Escherichia coli*). This modulation of the microbiota is directly linked to changes in metabolic products, including SCFAs, lipopolysaccharides (LPS), and trimethylamine N-oxide (TMAO), suggesting that the gut microbiota may underpin the neuroprotective effects of TCM.

**TABLE 1:** Traditional Chinese medicine regulates the composition and structure of gut microbiota.

TCM intervention	Formulation composition	Preparation method	Completeness of drug details in original studies	Experimental subjects	Changes in intestinal flora abundance	Changes of metabolites	Possible mechanisms	References
Banxia Xiexin Decoction	*Pinellia ternata, Radix scutellariae, Rhizoma coptidis, Rhizoma zingiberis, Radix ginseng, Radix Glycyrrhizae Preparata* and *Fructus jujubae* Weight Ratio: 3:2:1:2:2:2:3	Boil-free granules were dissolved in 0.5% carboxymethyl cellulose	Full details reported	APP/PS1 mice	*Probeobacteria*↓ *Deferribacterales*↓ *Alistipes*↓ *Bacteroides*↓ Rikenellaceae↓	SCFAs↑ GLP-1↑ GLP-1R↑	Modulates gut microbiota, stimulates GLP-1/GLP-1R secretion, enhances PI3K/Akt/GSK3β insulin signaling pathway, and improves cerebral glucose metabolism	Gao et al. (2023)
Chaihu Shugan San	*Bupleurum chinensis DC., Citrus reticulata Blanco, Paeonia lactiflora Pall, Ligusticum chuanxiong Hort., Cyperus rotundus L., Citrus aurantium L.* and *Glycyrrhiza uralensis Fisch* Weight Ratio: 4:4:3:3:3:3:1	Water extract (boiling distilled water, reflux, 2h)	Full details reported	SAMP8 mice	*S. xylosus*↓ *L. reuteri*↑	-	Reduces neuronal damage and neuroinflammation by altering gut microbiota in aged mice	Li et al. (2023c)
Qi-Fu-Yin	*Ginseng, Rehmannia glutinosa, Angelicae Sinensis, Atractylodes macrocephala, Polygala tenuifolia, Glycyrrhiza* (Prepared) and *Spina Date Seed*	Traditional water decoction	Some details (Composition and Preparation)	APP/PS1 mice	Erysipelotrichaceae↑ *Bacteroides*↓ Rikenellaceae↓	-	Regulates bacteria related to pathophysiology and cognitive impairment in APP/PS1 transgenic mice, improving KEGG pathways, carbohydrate-active enzymes, and virulence factors in the gut microbiota	Xiao et al. (2022)
CA-30, an oligosaccharide fraction derived from Liuwei Dihuang decoction	CA-30 component is mainly composed of stachyose and mannotriose	Extracted from the Liuwei Dihuang decoction	Full details reported	SAMP8mice	*Bacteroides*↓ *Parabacteroides*↓ *Acidiphilium*↑ *Prevotella*↑ *Ruminococcus*↓	gonadotropin-releasing hormone (GnRH)↓ luteinizing hormone (LH)↓ testosterone (T)↓ thyrotropic hormone-releasing hormone (TSH)↑ growth hormone (GH)↑	Improve the intestinal microbiome, rebalance the NIM network, and improve AD cognitive dysfunction	Wang et al. (2019a)
Epigallocatechin-3-gallate (EGCG)	-	Commercial channel acquisition (Sigma, catalog #E4143)	Full details reported	HFD-fed estrogen-deficient mice	*Prevotella*↓ *Bifidobacteriales*↑ *Verrucomicrobia*↑ *Actinobacteria*↓ Porphyromonadaceae↓ Bacteroidellaceae ↑ Rikenellaceae↑ *Olsenella*↓	-	Modulates key bacteria linked to cognitive deficits, reduces inflammation and oxidative stress, and regulates iron-related transport proteins	Qu et al. (2022b)
Ginsenoside Rg1	-	Commercial channel acquisition (KPC Pharmaceuticals, Inc. purity≥98%; Kunming, China)	Full details reported	D-gal + Aβ25-35 induced tree shrews	*Lactobacillus*↑ *Proteobacteria*↑ *Verrucomicrobia*↑	-	Restores gut microbiota composition and abundance, reducing tau protein	Wang et al. (2020a)
Ginsenoside Rg1	-	Commercial channel acquisition (Kunming Pharmaceutical Group Co., Ltd.)	Full details reported	D-gal + Aβ25-35 induced tree shrews	*Bacteroidetes*↓ *Lactobacillus salivarius*↑	-	Improves gut microbiota, inhibits pro-apoptotic proteins, and reduces Aβ and tau phosphorylation	Guo et al. (2021)
Silibinin and Silymarin	-	Commercial channel acquisition (Sigma-Aldrich Co., Shanghai, China)	Full details reported	APP/PS1 mice	*Verrucomicrobia*↑ *Akkermansia*↑ *Allobaculum*↑ *Butyricicoccus*↑	D-alanine ↑	Alters gut microbiota structure and abundance, influencing specific AD-related metabolic pathways	Shen et al. (2019)
Quercetin	-	Commercial channel acquisition (Q4951-10G, Sigma-Aldrich, United States)	Full details reported	Aged ICR mice induced by short-term dietary intake of advanced glycation end products	*Tenericum*↑ *Verrucommicrobia*↓ *Blautia*↓ *Anaerotruncus*↓	-	Increases gut microbial α-diversity, reduces tau protein phosphorylation and neuroinflammation	Yang et al. (2020)
Quercetin	-	Commercial channel acquisition (Q4951-10G, Sigma-Aldrich, United States)	Full details reported	APP/PS1 mice	*Facklamia*↑ *Aerococcus*↑ *Glutamicibacter*↑	BDNF↑ miR-26a↓ miR-132↑	Enhances gut microbiota diversity, reduces Aβ plaques, tau phosphorylation, and neuroinflammation	Lv et al. (2018)
Total saikosaponins	-	Radix Bupleuri (the Chinese Medicinal Material market in Bozhou, China, Lot No. 20190311). 80% EtOH (pH9) reflux →AB-8 resin (70% EtOH elution) → freeze-dried	Full details reported	APP/PS1 mice	*Desuflovibrio*↓ *Helicobacter*↓ *Mucispirillum*↓ *Roseburia*↓ *Clostridium*↓	-	Modulates gut microbiota to regulate metabolism, inhibit inflammatory factors release, and reduce Aβ deposition	Li et al. (2022b)
2H-gentiopicroside	-	Extracted from Dried Gentiana rigescens Franch (Huqingyutang Chinese Pharmacy Hangzhou, Zhejiang Province)	Full details reported	D-galactose-induced AD mice	*Firmicutes*↑ Lachnospiraceae↑ Ruminococcaceae↑ Peptococcaceae↑ *Bacteroidetes* ↓ Veillonellaceae↓	-	Reshapes gut microbiota, promotes beneficial bacterial abundance, inhibits pro-inflammatory bacteria, and alleviates neuroinflammation	Wang et al. (2023a)
Ginkgolide B	-	Commercial channel acquisition (Yuanye Biotechnology Co., Ltd., Shanghai, China)	Full details reported	Mice induced by D-galactose and aluminum chloride	*Lactobacillus*↑ *Firmicutes*↑ *Bacteroidales*↓ Muribaculaceae↓ *Alloprevotella*↓	SCFAs↑	Reverses abnormal gut microbiota, exerts partial neuroprotection, and reduces RAGE levels and Bax/Bcl-2 ratio	Liu et al. (2021)
Gastrodin	-	Commercial channel acquisition (Shanghai Aladdin Biochemical Technology Co., Ltd)	Full details reported	APP/PS1 mice	*Firmicutes*↓ Staphylococcaceae↓ *Staphylococcus*↓ *Bacteroidota*↑ *Actinobacteriota*↑	Bcl-2↑ Bax↓ IGF-1 ↑ CREB↑	Modulates abnormal gut microbiota, regulates the IGF-1 pathway, and inhibits neuronal apoptosis through the gut-brain axis	Zhang et al. (2023b)

IGF-1: Insulin-like Growth Factor 1, ↓:downregulation or inhibition; ↑: upregulation or activation.

Banxia Xiexin Decoction is essential in the initial regulation of the gut microbiota in AD treatment. It reshapes the gut microbiota structure and activates the GLP-1/GLP-1R pathway, thereby reducing amyloid deposition, enhancing synaptic plasticity, and alleviating cognitive dysfunction (Gao et al., 2023). In this process, modulation of the gut-brain axis is considered the basis of its neuroprotective mechanism. Additionally, a study by Li et al. investigated the impact of Chaihu Shugan Powder on the gut microbiota-gut-brain axis in SAMP8 mice. They found that changes in the abundance of specific bacteria, such as *Lactobacillus reuteri*, were closely correlated with cognitive performance in the mice, further highlighting the importance of gut microbiota in the anti-AD effects of TCM (Li Z. et al., 2023). Research on the anti-aging mechanisms of Qi-Fu-Yin also supports this (Xiao et al., 2022), demonstrating that the formula exerts anti-AD effects by regulating the diversity and species composition of the gut microbiota, as well as the function of key active enzymes. The oligosaccharide metabolite CA-30 derived from Liuwei Dihuang Decoction also shows promise in AD treatment. By improving microbial diversity and modulating Carbohydrate-Active enZymes (CAZymes) - key microbial enzymes involved in complex carbohydrate breakdown and modification - in the gut microbiome, CA-30 can regulate the gut-brain axis, delay the aging process, and enhance cognitive function. Specifically, CA-30 administration notably reversed deficits in the glycoside hydrolase family GH85, which is implicated in cleaving potentially neurotoxic GlcNAc polymers associated with AD pathology. Furthermore, CA-30 plays a significant role in regulating the secretion of hormones and cytokines in the NIM network (Wang J. et al., 2019).

Moreover, many TCM extracts and active metabolites have demonstrated notable effects in modulating the gut microbiota and improving cognitive function. For instance, EGCG has been shown to improve cognitive deficits in estrogen-deficient mice fed a high-fat diet (HFD) by regulating Prevotella and Bifidobacterium (Qu Y. et al., 2022). GRg1 has been found to alleviate AD symptoms, with changes in the abundance of Proteobacteria and Verrucomicrobia closely associated with this effect (Wang L. et al., 2020). Silymarin and silybin have been shown to regulate the abundance of key bacterial species linked to AD, thereby improving memory and histological parameters in APP/PS1 mice (Shen et al., 2019). Research by Lv et al. demonstrated that, under low vitamin D conditions, quercetin significantly enhanced gut microbiota diversity, which in turn improved cognitive function (Lv et al., 2018), further highlighting the close relationship between TCM, gut microbiota, and brain function.

### 7.2 Enhancement of immune function

The relationship between gut microbiota and the immune system plays a central role in the pathological mechanisms of AD. TCM modulates the balance of the gut microbiota, influencing not only gut immune functions but also brain immune responses (see Table 2).

**TABLE 2:** Traditional Chinese medicine affects immune function by regulating gut microbiota.

TCM intervention	Formulation composition	Preparation method	Completeness of drug details in original studies	Experimental subjects	Changes in intestinal flora abundance	Changes of metabolites	Possible mechanisms	References
Bushen Yinao pill	pilose antler (depilated), red ginseng, cooked Rehmannia, Chinese wolfberry, psoralen (salt), *Angelica sinensis, Ligusticum chuanxiong, Achyranthes bidentata,* Ophiopogon, Schisandra*,* sour jujube kernel (fried), cinnabar (Shuifei), *Poria*, Polygala, Radix scrophulariae, and fried yam	Prepared pills	Full details reported	AD patients	*Bifidobacterium*↑ *Lactobacillus*↑ *Bacteroides*↑ *Digestive cocci*↓ *Enterococci*↓	-	Increases beneficial gut bacteria, reduces harmful bacteria, decreases levels ofinflammatory markers such as Aβ, IL-6, and TNF-α, and significant improve cognitive function	Wang et al. (2025)
Kai-xin-san	*Ginseng Radix et rhizoma, Poria, Polygalae radix* and *Acori tatarinowii rhizoma*	Dry powder	Some details (Composition and Preparation)	D-gal + Aβ25-35 induced rats	*Lactobacillus_murinus*↑ *Ligilactobacillus*↑ *Alloprevotella*↑ *Pre votellaceae_NK3B31_group*↑ *Allobaculum* ↓ *Clos tridium_sensu_stricto_1*↓	-	Restores gut microbiota homeostasis, alleviates gut inflammation, reverses dysbiosis, reduces neuroinflammatory response in the brain, and improves cognition	Wang et al. (2024a)
Jiedu Yizhi Formula	*Coptidis Rhizoma, Alpiniae Oxyphyllae Fructus, Carapax et Plastrum Testudinis Colla, Rhei Radix et Rhizoma, Pheretima, Corni Fructus* and *Chuanxiong Rhizoma*. (Jilin Hongjian Pharmacy Co., Ltd., Jilin, China) Weight Ratio: 1:2:1:1:1:1:1	Boiled and then prepared into freeze-dried powder	Full details reported	APP/PS1 mice	Ruminococcaceae↑ *Actinobacteria*↑ *Alistipes*↓ *Lachnospiracea*↓ *Deferribacteres*↓ Muribaculaceae↓	SCFAs↑	Enhances gut microbiota diversity and composition, inhibits TLR4/NF-κB signaling in the brain immune system, modulates oxidative stress, reduces glial activation, and reverses p-tau phosphorylation	Zhang et al. (2023a)
Tetragonia tetragonioides	*-*	Extracted from Tetragonia tetragonioides Kuntze (grown in Jeju Island) 70% EtOH or H_2_O, 25°C/24h shaking → 8000×g/30min centrifugation → freeze-dried	Full details reported	Aβ25-35-induced high-fat diet in AD rats	*Erysipelotrichales*↓ *Clostridiales*↓ *Desulfovibrionales*↓ *Enterobacteriales*↓ *Bacteroidales*↑ *Lactobacillales*↑	-	Elevates Hippocampal Amyloid-β Deposition through Potentiating Insulin Signaling and Altering Gut Microbiome Composition	Kim et al. (2020)
Gegen Qinlian tablets	-	Commercial tablet (CSPC Pharmaceutical Group Co., Ltd., Shijiazhuang, China, No. Z45020815)	Some details (Preparation)	AD rats induced by Aβ1-42	Prevotellaceae↓ *Prevotella*↓ Ruminococcaceae↑ *Oscillospira*↑ *Ascomycota*↑ Aspergillaceae↑ *Aspergillus*↑	Tnfaip2↓ phosphorylated-p38, ERK, and JNK↓	Restores abnormal gut bacterial and fungal homeostasis, reduces neuroinflammation, and improves cognitive function	Wang et al. (2024b)
Scutellarin	-	Commercial channel acquisition (lot number: 20161003, purity 160 ≥ 90%, Yunnan Bio Valley, China.)	Full details reported	APP/PS1 mice	*Paraprevotella*↑ *Alloprevotella*↑ *Parabacteroides*↓ *Parasutterella*↓ Desulfovibrionaceae↓ *Dubosiella*↓	Acetate in feces↓	Modulates gut microbiota and microglial cAMP-PKA-CREB-HDAC3 signaling to reverse neuroinflammation and cognitive impairment in APP/PS1 mice	Zhang et al. (2022c)
Patchouli alcohol	-	Iisolated and purified in the laboratory of Prof. Ziren SU at Guangzhou University of Chinese Medicine, Guangdong Province, China	Full details reported	TgCRND8 transgenic AD mouse	Lactobacillaceae↓ Enterobacteriaceae↓ Erysipelotrichaceae↑ *Clostridium*↑ *Alistipes*↓ *Bacteroides*↓ *Bilophila*↓ *Blautia*↓	-	Inhibits the C/EBPβ/AEP signaling pathway activation, regulating gut microbiota and exerting anti-AD effects	Xu et al. (2023)
Curcumin	-	Commercial channel acquisition (Sigma-Aldrich, St. Louis, MO, United States)	Full details reported	3xTg-AD mice	*Verrucomicrobia*↓ Oscillospiraceae↑ Rikenellaceae↑ *Oscillibacter*↑ *Alistipes*↑ *Pseudoflavonifractor*↑ *Duncaniella*↑ *Flintibacter*↑	-	Alters specific bacterial populations, improves metabolic function, reduces inflammation, and enhances memory function	Lamichhane et al. (2024)

↓: downregulation or inhibition; ↑: upregulation or activation.

A recent clinical study demonstrates the efficacy of Bushen Yinao pill in improving cognitive function in AD patients by regulating gut microbiota and reducing inflammation, with a favorable safety profile (Wang et al., 2025). Kaixin San (KXS) has been shown to regulate both gut and brain functions via the gut-brain axis. A study by Wang et al. demonstrated that KXS effectively restores gut microbiota balance and inhibits progressive inflammation through neural and immune pathways, thereby providing neuroprotective effects (Wang H. et al., 2024). Jiedu Yizhi Formula, on the other hand, modulates the gut microbiota, blocks the TLR4/NF-κB pathway, and inhibits inflammation, which mitigates cognitive impairments caused by inflammation and interrupts the vicious cycle of AD (Zhang P. et al., 2023). Notably, after 8 weeks of administration, no pathological signs of liver or kidney damage were observed, further supporting the formula’s safety. Moreover, *Tetragonia tetragonioides* has shown potential in regulating immune function by modulating the gut microbiota and improving insulin resistance. It reduces neuroinflammation, enhances insulin signaling pathways, and improves memory function (Kim et al., 2020). This mechanism involves both gut microbiota modulation and improvements in insulin metabolism and immune responses, further underscoring TCM’s multi-target therapeutic potential.

Recent studies have also highlighted the immune-modulating effects of various TCM components. Gegenqinlian Tablets, a modern TCM formulation, contain high concentrations of polar active metabolites, such as berberine and baicalein (Wang et al., 2016). These metabolites can improve gut dysbiosis and, by regulating the NF-κB/MAPK signaling pathway, inhibit inflammatory responses in the hippocampus while improving microglial morphology (Wang L. et al., 2024). Additionally, baicalein regulates the gut microbiota and SCFA levels, suppresses the cAMP-PKA-CREB-HDAC3 signaling pathway in microglia, reduces the expression of pro-inflammatory factors, alleviates neuroinflammation, and improves cognitive function in AD patients (Zhang S. et al., 2022). Xu et al. investigated the mechanism of patchouli alcohol (PA) in alleviating AD-related cognitive deficits and found that PA relieves gut microbiota imbalance, inhibits the C/EBPβ/AEP pathway, and reduces Aβ plaque deposition, tau hyperphosphorylation, and neuroinflammation (Xu et al., 2023).

Curcumin, a natural plant compound, has also demonstrated significant effects on immune function by modulating the gut microbiota. Studies show that curcumin improves gut microbiota composition in AD animal models induced by a high-fat, high-sugar diet, reduces inflammation, and positively impacts metabolic syndrome, as well as liver and cardiovascular systems (Lamichhane et al., 2024). Through interactions with both the gut-brain and gut-liver axes, curcumin further elucidates the integrated regulatory roles of TCM components across multiple physiological systems.

### 7.3 Modulation of endogenous products

TCM modulates the gut microbiota not only by altering its structure and function but also by potentially influencing the production of endogenous metabolites in the gut, thereby exerting neuroprotective effects. Recent research has increasingly highlighted the complex relationship between TCM, gut microbiota, and metabolites (see Table 3).

**TABLE 3:** Traditional Chinese medicine affects the production of endogenous metabolites in gut microbiota.

TCM intervention	Formulation composition	Preparation method	Completeness of drug details in original studies	Experimental subjects	Changes in intestinal flora abundance	Changes of metabolites	Possible mechanisms	References
Modified Huang-Lian-Jie-Du Decoction	*Rhizoma Coptidis, Cortex Phellodendri, Fructus Gardeniae, Salvia miltiorrhiza, Curcuma longa L.,* and *Acorus tatarinowii* Weight Ratio: 3 : 2: 3 : 3: 2 : 2	Water extract (boiling distilled water, reflux, 2h)	Full details reported	Aβ1-42-induced C57BL/6 mice	Rikenellaceae↑ *Oscillospira*↑ *Dorea*↑ *Bacteroides*↓ *Parabacteroides*↓ Mycoplasmataceae↓	Glutamate metabolism↑ Aspartate metabolism↑ Adenosine↑	Modulates gut microbiota composition and abundance, potentially involving NMDA receptor-mediated glutamatergic transmission and adenosine/ATPase/AMPK cascade	Liu et al. (2019)
Hawthorn flavonoid	-	Obtained (91.26% purity) from Qingyun-Bio Co., Ltd (Nanjing, china)	Full details reported	AD mice induced by D-galactose and aluminum chloride	*Dubosiella*↑ *Alloprevotella*↑ *Bifidobacterium*↑ *Acinetobacter*↓	Docosapentaenoic acid (DPA) ↑ Sphingolipid (SM) ↑ Phosphatidylcholine (PC) ↑	Corrects gut dysbiosis, regulates metabolic disorders, reduces oxidative stress, Aβ aggregation, and microglial activation, improving cognitive impairment	Zhang et al. (2022b)
Quercetin-3-O-Glucuronide	-	Commercial channel acquisition (WuXi Apptec Co., Ltd., China)	Full details reported	AD mice induced by Aβ1-42	*Barnesiella* ↑ *Lactobacillus*↑ *Alistipes*↓ *Rikenella*↓	SCFAs↑ BDNF↑	Restores gut microbiota, increases SCFAs, enhances CREB and BDNF levels, and alleviates Aβ accumulation and tau phosphorylation	Xu et al. (2021)
*Schisandra chinensis polysaccharide* (SCP)	*-*	Boiling H_2_O extraction →70% EtOH ppt. →Sevage deproteinization→ dialysis→ EtOH ppt. (SCP) → DEAE-52 [0.2M NaCl elution] → Sephadex G-100 (SCP2)	Full details reported	AD rat model induced by Aβ25-35	*Bacteroides*↑ *Coprococcus*↑ *Paraprevotella*↑	SCFAs↑ Oxidized glutaric acid ↑ Succinic acid ↑ Arachidonic acid ↓ Linoleic acid and α-linolenic acid ↑	Modulates fecal metabolites and gut dysbiosis, repairs gut barrier, and reduces neuroinflammation	Fu et al. (2023)
Walnut-Derived Peptide (PW5)	-	Derived from walnut (Shangluo City, southeast Shanxi province), chemically synthesized (purity > 99%) by GL Biochem Co., Ltd (Shanghai, China)	Full details reported	APP/PS1 mice	*Firmicutes*↑ *Proteobacteria*↓ *Verrucomicrobia*↓ *Norank_f_*Enterobacteriaceae↑ *unclassified_f_*Helicobacteraceae↑ *Allobaculum spp* ↑ *norank_f_*Erysipelotrichaceae↑ *unclassified_o_Bacteroidales_c_Bacteroidia*↑ *Flexispira spp*↑ *Mucispirillum spp*↓	norepinephrine (NE)↑ isovalerate↑ acetylcholine (AChe)↓ valerate↓	Changes in gut microbiota and serum metabolite composition and reduced Aβ plaques	Wang et al. (2019b)
Oligosaccharides from Morinda officinalis	-	Isolated from Morinda officinalis	Some details (Name and origin)	APP/PS1 mice	*Firmicutes*↑ *Bacteroidetes*↓ *Proteobacteria*↓ *Actinobacteria*↓ *Cyanobacteria*↓	Amino acid↓ Phosphatidylethanolamine (PE)↑ Lysophosphatidylethanolamine (LPE)↓ Phosphatidylcholine↑	Inhibits AD progression by modulating key microbiota-metabolite interactions	Xin et al. (2019)
Danggui Shaoyao San	*Angelica sinensis, Paeonia lactiflora, Poria cocos, Atractylodes macrocephala, Alisma orientalis* and *Ligusticum chuanxiong.* Weight Ratio: 3:16:4:4:8:8	Water extract. Purchased from the Chinese Medicine Pharmacy of the First Affiliated Hospital of Hunan University of Chinese Medicine (No.19615)	Full details reported	STZ-induced AD rats	*Lactobacillus_reuteri*↑ *Akkermansia*↑ *Helicobacter_pylori*↓	TMAO↓	Increases the expression of synaptophysin I and PSD95, reduces hippocampal Aβ levels, and protects neurons from damage	Jin et al. (2023)
GuanXinNing Tablet	-	Commercial tablet (Chiatai Qinchunbao Pharmaceutical Co. Ltd., Hangzhou, China, No. Z20150028)	Some details (Name and origin)	An AD model rabbit fed a 2% cholesterol diet	*Firmicutes/Bacteroidetes*↓ *Verrucomicrobia*↑ *Akkermansia*↑ *dgA-11_gut_group*↑	TMAO↓ SOD, Bcl-2↑ MDA, Bax↓ TC, LDL↓ Glutamic acid↑ Citric acid↑ 3-Hydroxybutyric acid↑ Leucine↑	Alleviates AD by improving gut microbiota, host metabolites, and neuronal apoptosis	Zhang et al. (2021a)
Rich in phenylpropanoids-polyacetylenes and polysaccharides from *Codonopsis Radix*	-	From *Codonopsis Radix*: (1) 60% EtOH reflux → D101 resin [90% EtOH eluate] (EPP); (2) Boil H_2_O extr. → 80% EtOH ppt. → centrif. (POL)	Full details reported	SCOP-induced AD mouse model	*Colidextribacter*↓ *Lactobacillus*↓ *Bacteroides*↓ *Muribaculum*↓ *Roseburia*↑ Lachnospiraceae*_NK4A136_group*↑	LPS↓ occludin↑ acetylcholine (AChE)↓ Choline Acetyltransferase (ChAT)↑ BDNF↑	Modulates gut microbiota, repairs gut mucosa, reduces LPS entry into circulation, alleviating chronic inflammation and preserving cognitive function	Xie et al. (2024)

↓:downregulation or inhibition; ↑: upregulation or activation. EtOH: ethanol; EEP: extract rich in phenylpropanoids-polyacetylenes; POL: polysaccharides;

Modified Huanglian Jiedu Decoction (MHJD) has been shown to influence the adenosine pathway through gut microbiota modulation. This effect may be mediated by NMDA receptor-dependent glutamatergic transmission (Liu et al., 2019). These findings suggest that the gut microbiota not only modulates immune and inflammatory responses but may also contribute to the neuroprotective mechanisms of AD through the regulation of neurotransmitter metabolism. A similar observation was made in studies on hawthorn flavonoids. Zhang et al. demonstrated that hawthorn flavonoids significantly improved cognitive deficits in AD mice by increasing the abundance of *Bifidobacterium* and enhancing the metabolism of docosapentaenoic acid (DPA) (Zhang J. et al., 2022). This suggests that specific changes in gut microbiota composition and metabolite production are closely linked and play a crucial role in the progression of AD.

Additionally, quercetin and its glucuronide conjugate (Q3G) have been shown to increase the production of SCFAs, restore gut microbiota balance, and reverse neuroinflammation and brain insulin resistance through the gut-brain axis (Xu et al., 2021). Polysaccharides in the botanical drug *Schisandra chinensis* have also been found to improve the gut microbiota and positively influence endogenous metabolite production, exerting neuroprotective effects by promoting the generation of unsaturated fatty acids (Fu et al., 2023). Walnut-derived peptide PW5, an active metabolite of walnut protein hydrolysates, has demonstrated its ability to improve cognitive function in APP/PS1 mice by altering the gut microbiota and serum metabolites (Wang M. et al., 2019). Research on PW5 further supports the potential of TCM in combating AD by influencing gut microbiota and metabolites, particularly in terms of reducing Aβ42 aggregation and improving cognitive function.

In studies focusing on specific metabolites, Xin et al. examined the modulation of gut microbiota and metabolites by oligosaccharides from *Gynochthodes officinalis* in AD mice. They identified six key “microbiota-metabolite” pairs and revealed a close relationship between gut microbiota and amino acid, fatty acid, and phospholipid metabolism (Xin et al., 2019). This finding provides more powerful experimental evidence for the role of TCM in regulating gut microbiota and its metabolites in neuroprotection.

### 7.4 Regulation of specific metabolic pathways

TCM plays a crucial role in treating AD by regulating the gut microbiota and influencing metabolic processes, particularly through the modulation of specific pathways that slow the pathological progression of AD. These mechanisms primarily involve the regulation of neurotransmitters, amino acid pathways, fatty acid metabolism, and bile acid regulation, among other metabolic pathways. Studies have demonstrated that various TCM botanical drugs and their active metabolites can specifically modulate the gut microbiota and its metabolites, thereby improving the pathological features of AD (see Table 4).

**TABLE 4:** Traditional Chinese medicine affects metabolic processes by regulating the gut microbiota.

TCM intervention	Formulation composition	Preparation method	Completeness of drug details in original studies	Experimental subjects	Changes in intestinal flora abundance	Changes of metabolites	Possible mechanisms	References
Danggui Shaoyao San	*Angelica sinensis, Paeonia lactiflora, Poria cocos, Atractylodes macrocephala, Alisma orientalis* and *Ligusticum chuanxiong*. (The Traditional Chinese Medicine Pharmacy at the First Affiliated Hospital of Hunan University of Chinese Medicine.) Weight Ratio: 3:16:4:4:8:8	Water extract	Full details reported	STZ-induced AD rats	*Ligilactobacillus*↑	Ophthalmic acid↓ Phosphocreatine↑	Modulates inflammation and oxidative stress	He et al. (2023)
*Rheum tanguticum*	*-*	Water extract (ZISUN MEDICINE HEALTH CO.LTD. GuangZhou, China, No.171202)	Full details reported	APP/PS1 mice	*norank_f_*Ruminococcaceae↑ *Erysipelatoclostridium*↑ *Bacteroides*↑ *Marvinbryantia*↓	Phosphatidylcholine↓ LysoPE↑ Glutamate↑ Pyroglutamate↑ 3-hydroxyundecanoyl carnitine↓ o-tyrosine↓	Alleviates cognitive dysfunction by modulating drug-responsive bacteria and their corresponding microbial metabolites	Gao et al. (2021)
Baicalein	-	Commercial channel acquisition (Sigma-Aldrich Chemical Corporation, St. Louis, MO, United States)	Full details reported	APP/PS1 mice	*g_Lactobacillus*↑ *g_Bifidobacterium*↑ *g_Tritrichomonas*↑ *g_Clostridium*↑ *s_Eubacterium Plexicaudatum*↓ *s_*Lachnospiraceae *bacterium 3–2*↓ *s_Dorea SP 5–2*↓ *s_Ruminococcus_sp_1xD21-23*↓	Glutamic acid ↑ Thymine ↑ caproyl coenzyme A ↑ Glycerophospholipid ↑	Treats AD by reshaping gut microbiota and metabolites	Shi et al. (2023)
*Poria cocos*	*-*	Water extract. (From Yunnan, China. Stored in Changchun Institute of Applied Chemistry, No. 20180321)	Full details reported	APP/PS1 mice	*Firmicutes*↓ *Bacteroidetes*↓ *Proteobacteria*↓ *Cyanobacteria*↓ Bacteroidaceae↓ Lachnospiraceae↓ Ruminococcaceae↓ Rikenellaceae↓ Deferribacteraceae ↓ Muribaculaceae↑ Lactobacillaceae↑ *Enterobacteriales*↓	LCA/CDCA↑ DCA/CA↓	Improves Gut Flora Dysbiosis, Reversing Bile Acid Metabolism Dysfunction, and Increasing Aβ Clearance	Sun et al. (2021)
Schisandrin	-	Commercial channel acquisition (purity >98%, the National Institute for Food and Drug Control)	Full details reported	D-gal + Aβ25-35 induced rats	*Desulfovibrio*↓ Christensenellaceae↑ Ruminococcaceae↑ *escherichia-Shigella*↓	Indole acrylic acid ↑ Cholic acid ↑ Sphingolipid ↑ Docosahexaenoic Acid (DHA)↑ Oleic acid, linoleic acid ↑ Palmitic acid ↓ DGLA, stearic acid ↑ Arachidonic acid ↓	Improves gut microenvironment, corrects gut-brain axis metabolism, reduces inflammation, and repairs barrier damage	Zhang et al. (2022a)
Icariin	-	Commercial channel acquisition (≥98% purity, Nanjing Spring and Autumn Biological Engineering Co., Ltd., CH33H40015)	Full details reported	APP/PS1 mice	*Akkermansia*↑ *Alistipe*↓	Glycerophospholipid ↑ Sphingolipid ↑Ceramide↓	Regulates gut microbiota involved in sphingolipid metabolism, improves gut barrier, and reduces Aβ deposition, neuroinflammation, and neuronal death	Liu et al. (2023)
Icariin and Panax notoginseng Saponins	-	Icariin (F1806075, Aladdin, Shanghai, China) and Panax notoginseng Saponins (1019C021, Solarbio, Beijing, China)	Full details reported	APP/PS1 mice	*Lactobacillus*↑ *Bifidobacterium*↑ *Adlercreutzia*↑ *Bacteroides*↓ *Paraprevotella*↓	MIPT3↓ Oasl1↑ TCHP↑	Improves gut microbiota distribution and regulates Oasl1, TCHP, and MIPT3 expression	Zhang et al. (2020)
Ginkgo biloba Extract	It consists of 44% flavonoids and 6% lactones	From SPH XingLing Sci and Tech. Pharmaceutical Co., Ltd. (Shanghai, China, approval number Z20000049)	Full details reported	APP/PS1 mice	*Bifidobacterium_pseudolongum*↑ *Limosilactobacillus_reuteri*↑ *Turicibacter_*sp.*_TS3*↑ *oriobacteriaceae_bacterium*↑ *dlercreutzia_caecimuris*↑ *kkermansia_muciniphila*↑	Tryptophan ↑ Neurosteroids ↑ Neuroactive ligand-receptor ↑	Modulates gut microbiota diversity and abundance, regulates tryptophan metabolism, steroid hormone biosynthesis, and neuroactive ligand-receptor interaction to alleviate AD pathology	Yu et al. (2023)
P-coumaric acid	-	Commercial channel acquisition (purity ≥98%, S31136, Yuanye Bio-Technology Co., Ltd, Shanghai, China)	Full details reported	AD mice induced by Aβ25-35	*Anaeroplasma*↓ *morganella*↓ *acetatifactor*↓ *helicobacter*↓ *serratia*↓ *holdemanella*↓ *ASF356*↓ *cobetia*↓ *fusicatenibacter*↓ *tannerellaceae unclassified*↓ *candidatus saccharimonas*↑ *clostridium sensu stricto 13*↑ *leuconostoc*↑ *gastranaerophilales unclassified*↑	Arachidonic acid↓ Glut1↑	Corrects gut dysbiosis and serum metabolite changes, reducing neuroinflammation and oxidative stress	Cao et al. (2023)

LCA: lithocholic acid, CDCA: Chenodeoxycholic Acid, DCA: deoxycholic acid, CA: cholic acid, DGLA: Dihomo-γ-linolenic Acid, MIPT3: Macrophage Inflammatory Protein 3, Oasl1: 2′,5′-oligoadenylate synthetase-like 1, TCHP: Total Cholesterol and High-Density Lipoprotein Cholesterol,↓:downregulation or inhibition; ↑: upregulation or activation.

Danggui Shaoyao San has been shown to restore the balance between *Candida* and OA interactions, modulating purine and nicotinic acid-nicotinamide metabolism, which helps alleviate brain inflammation and oxidative stress (He et al., 2023). *Rheum palmatum* regulates specific bacterial species, such as *Marvinbryantia*, influencing neurotransmitter and amino acid metabolic pathways. Notably, the reduction of O-tyrosine in the gut may be a key target for its improvement of cognitive dysfunction (Gao et al., 2021). Baicalein further confirms this mechanism, affecting glutamate and glycerophospholipid metabolism through the regulation of the gut microbiome, thereby improving the learning and memory abilities of APP/PS1 mice (Shi et al., 2023). Poria cocos (Fuling), by regulating gut microbiota balance, can reverse bile acid metabolic dysfunction (Sun et al., 2021). Schisandrin, found in *S. chinensis*, although having low oral bioavailability, significantly impacts primary bile acid biosynthesis and sphingolipid metabolism (Zhang C. et al., 2022). Studies on icariin reveal the importance of sphingolipid metabolism in AD treatment, as it, in combination with Panax notoginseng Saponins, significantly increases bacterial diversity, modulates protein expression, and inhibits the release of inflammatory factors (Liu et al., 2023; Zhang et al., 2020).

*Ginkgo biloba* extract has been shown to enhance the presence of beneficial bacteria, modulate levels of SCFAs, tryptophan, and steroid hormones, and restore synaptic plasticity (Yu et al., 2023). The study of p-coumaric acid (PCA) further supports the significance of energy metabolism. PCA restores the abundance of specific bacterial species, modulates glucose and arachidonic acid metabolism, and affects MAPK/NF-κB and PI3K/AKT/Glut1 signaling pathways, thereby exerting anti-AD effects (Cao et al., 2023).

These findings indicate that TCM regulates the gut microbiota and its metabolic processes through multiple targets and pathways, playing a significant role in AD treatment. This offers new insights for developing AD therapeutic strategies based on the “microbiota-metabolite-brain axis.”

### 7.5 Repair of barriers

Research has shown that damage to the intestinal barrier is closely linked to the onset of AD, and gut microbiota dysbiosis may be a key factor contributing to increased intestinal permeability, exacerbated neuroinflammation, and amyloid-beta (Aβ) accumulation. Therefore, restoring intestinal barrier function and modulating gut microbiota composition have become crucial mechanisms through which TCM contributes to the prevention and treatment of AD (see Table 5).

**TABLE 5:** Traditional Chinese medicine repairs the intestinal barrier by regulating the gut microbiota.

TCM intervention	Formulation composition	Preparation method	Completeness of drug details in original studies	Experimental subjects	Changes in intestinal flora abundance	Changes of metabolites	Possible mechanisms	References
Korean Red Ginseng	-	Water extract. (Korea Ginseng Corporation, Daejeon, Korea)	Full details reported	Tg2576 heterozygous transgenic mice	*Lactobacillus*↑ *Bifidobacterium*↓ *Ruminococcus*↓ *Clostridia*↓	Claudin-5↑ Occludin↑ Laminin↑	Reduces Hippocampal Amyloid-β Deposition through Potentiating Insulin Signaling and Altering Gut Microbiome Composition	Lee et al. (2022b)
*Polygonatum sibiricum polysaccharides*	*-*	From P. sibiricum rhizomes: DEAE-52 → Sephacry-200 [0.1M NaCl elution, UV490 nm] → PSP-1	Full details reported	5xFAD mice	*Helicobacter typhlonius*↓ *Helicobacter masomyrinus*↓ *Akkermansia muciniphila*↑	-	Reshapes gut microbiota, alleviates gut barrier damage, inflammatory response, and gut Aβ deposition	Luo et al. (2022)
Sinomenine	-	Sinomenine (SIN, #S27281) was acquired from Shanghai yuanye Bio-Technology (HPLC purity ≥98%, Shanghai, China)	Full details reported	SCOP-induced AD mouse model	*Odoribacter*↑ *Mucispirillum*↑ *Bacteroides/Firmicutes*↑ Muribaculaceae↑ *Bifidobacterium*↑ *Bacilli*↓(except for *Bacteroidetes*)	GDNF↑ BDNF↑ ACh、ChAT↑ acetylcholine (AChE)↓ α7nAChR↓ LPS↓	Corrects gut dysbiosis, exerts anti-inflammatory, gut mucosal barrier protection, and neuroprotective effects, restores cholinergic balance, and preserves gut-brain axis homeostasis	Ni et al. (2024)
Berberine	-	Not specified	Minimal details (only common name and dose)	5xFAD mice	*Enterococcus*↑ *Ligilactobacillus*↑ *Akkermansia*↑ *Roseburia*↑ *Lachnoclostridium*↑ *Paraprevotella*↓ *Alloprevotella*↓ *Bacteroide*↓	ZO-1↑ occludin↑ claudin-1 proteins↑	Improves gut microbiota composition, alleviates gut inflammation, reduces gut permeability, clears Aβ plaques, and increases neuronal survival	Sun et al. (2024)
Oligosaccharides From Morinda officinalis	-	From Morinda officinalis	Some details (Name and origin)	APP/PS1 mice	*Lactobacillus*↑ *Allobaculum*↑ Lactobacillaceae↑ Lachnospiraceae↑ *Akkermansia*↓ *Bacteroides*↓ *Roseburia*↓ *Bifidobacterium*↓ *Lactobacillus*↓ *Desulfovibrio*↓	Phosphatidylcholine↑ Phosphatidylethanolamine (PE)↑ Linoleic acid ↑ Diethyl phosphate ↑	Regulates neurotransmitter and neuromodulator production and secretion by improving gut microbiota	Xin et al. (2018)
Fructooligosaccharides from Morinda officinalis	-	From Morinda officinalis	Some details (Name and origin)	D-galactose and Aβ1-42-induced AD rats	*Lactobacillus*↑ *Akkermansia*↑ *Bifidobacteria*↑	norepinephrine (NE)↑ Dopamine (DA)↑ 5-HT↑ 5-HIAA↑	By regulating the gut microbiota, it can affect the host’s inflammatory immune response and oxidative stress, and regulate neurotransmitters to reduce neuronal apoptosis	Chen et al. (2017)
Gastrodin	-	From Gastrodia elata rhizome (Sichuan, voucher 20191114): 100% MeOH extr. → silica gel [CH_2_Cl_2_/MeOH 75:25] → prep-HPLC [5% aq. MeOH, tR = 41min] (Gas)	Full details reported	D-galactose-induced AD mice	Erysipelotrichaceae↑ Bacteroidaceae↑ Rhodospirillaceae↑ Tannerellaceae↑ Atopobiaceae↑ *Clostridiales_vadinBB60_ group*↓ Helicobacteraceae↓ *Lactobacillus*↑ *Firmicutes*↑ Muribaculaceae↑ *Verrucomicrobiae*↑	GABA↑ Acetylcholine↑ histamine↑ Palmitate↑	Modulates gut microbiota composition, maintains gut barrier and BBB function, ultimately enhancing cognition and neuroprotection	Fasina et al. (2022)

5-HT: 5-Hydroxytryptamine (Serotonin), 5-HIAA: 5-Hydroxyindoleacetic Acid,↓:downregulation or inhibition; ↑: upregulation or activation.

Korean red ginseng (*P. ginseng*) has been shown to reduce microglial activation and restore blood-brain barrier integrity by improving the gut microbiota structure, particularly by increasing the dominance of *lactobacilli*. This, in turn, helps reduce Aβ accumulation and improves cognitive and memory functions (Lee M. et al., 2022). Similarly, polysaccharides from *R. japonica* have demonstrated potential in intestinal barrier repair. Studies indicate that these polysaccharides inhibit harmful bacteria that accelerate AD progression while promoting beneficial microbial species, thereby strengthening the intestinal barrier function. Specifically, they increase the expression of occludin and ZO-1, reduce intestinal permeability, and improve the intestinal inflammatory environment by promoting goblet cell proliferation, effectively alleviating cognitive deficits in AD (Luo et al., 2022). Furthermore, sinomenine (SIN) plays a significant role in intestinal barrier repair by regulating gut-brain axis homeostasis and the cholinergic anti-inflammatory pathway (CAP). SIN not only improves gut microbiota dysbiosis but also restores the intestinal barrier by increasing the expression of ZO-1, occludin, and BDNF, thereby reducing neuroinflammation in both the gut and the brain. Its mechanisms of action also include modulating α7nAChR receptors and inhibiting the TLR4/NF-κB signaling pathway, further supporting its potential in AD prevention and treatment (Ni et al., 2024).

A recent study (Sun et al., 2024) revealed that berberine alleviates AD by modulating the gut microenvironment, restoring the intestinal barrier, and rebalancing the gut-brain axis. This likely results in the direct or indirect reduction of Aβ plaques and neuroinflammation, thereby promoting neuronal survival in the brain. Notably, Fructooligosaccharides from *G. officinalis*, as potential prebiotics, have demonstrated significant regulatory effects on the gut-brain axis by improving gut morphology, promoting mucin production, and reducing intestinal permeability (Chen et al., 2017; Xin et al., 2018).

In conclusion, TCM, through the regulation of the gut microbiota, not only helps repair the intestinal barrier and alleviate inflammation caused by increased intestinal permeability, but also effectively modulates the gut-brain axis, thereby positively impacting the prevention and treatment of AD.

### 7.6 Influence of the gut microbiota on the transformation of traditional Chinese medicine

Research indicates that the active components of TCM can exert anti-AD effects through interactions with the gut microbiota. In this context, the gut microbiota not only serves as a target for drug action but also participates in the metabolic conversion of these drugs. Although studies in this area are still limited, the anti-AD mechanisms of curcumin, a key active metabolite in *Curcuma longa*, *Curcuma aromatica*, and *Acorus calamus*, have been preliminarily elucidated. Sun ZZ et al. found that curcumin exerts anti-AD effects through a dual mechanism: on the one hand, it directly modulates the diversity of the gut microbiota in APP/PS1 mice, restoring the abundance of key microbiota associated with AD progression; on the other hand, the gut microbiota metabolizes curcumin into more potent anti-AD metabolites (Sun et al., 2020). This “microbiota-drug” interaction significantly enhances curcumin’s therapeutic effects. Notably, some TCM components, despite poor direct absorption, can still exert therapeutic effects by modulating the gut microbiota. For instance, Xanthoceraside (XAN), despite its poor solubility and low permeability, is almost entirely unabsorbed through the gastrointestinal tract. However, Zhou H’s study confirmed that XAN exerts anti-AD bioactivity via gut-brain metabolic pathways by significantly altering the structure of the gut microbiota (GM) (Zhou et al., 2019). This finding offers new insights into the mechanisms of TCM components with low bioavailability.

Nevertheless, several limitations persist in the current research. First, the specific molecular mechanisms underlying the interaction between TCM and the microbiota remain insufficiently explored. Second, the rules governing microbiota-mediated drug metabolism are not yet fully understood. Additionally, the influence of individual differences in microbiota composition on drug efficacy requires further investigation. Future research should focus on the role of the gut microbiota in TCM’s multi-component systems and the complex interaction network between the microbiota, host, and drugs, to provide more evidence supporting the scientific rationale behind TCM’s anti-AD effects.

## 8 Discussion and conclusion

Research on the modulation of gut microbiota in AD remains in its early stages. Studies primarily rely on 16S rRNA and metagenomic sequencing technologies to analyze changes in gut microbiota, alongside metabolomics to observe the distribution and effects of microbial metabolites in the body. Comparative analyses using germ-free and gnotobiotic animal models, as well as fecal microbiota transplantation and *in vitro* bacterial culture methods, have helped minimize experimental interference, making some progress. However, current research on microbial community function predominantly focuses on species composition and diversity, while overlooking their dynamic roles at the transcriptomic level.

To address these gaps, Shen Y et al. developed a droplet-based single-microbe RNA sequencing technology (smRNA-seq), enabling the construction of bacterial transcriptomic landscapes of the human gut microbiota at the single-cell level. This provides new tools for understanding the complex microbiota and its interactions with the host (Shen et al., 2024). Additionally, Vercauteren S introduced a CRISPR-based high-throughput genomic screening method, expanding the technical capabilities for microbial function studies (Vercauteren et al., 2024). As microbial detection technologies continue to improve, the mechanisms by which TCM modulates gut microbiota to treat AD will become clearer.

Studies have confirmed that changes in gut microbiota contribute to the pathogenesis of AD, contributing to a two-way regulatory process via the gut-brain axis. However, existing research has primarily focused on how gut microbiota influences the development of AD. In terms of treatment, interventions such as probiotics, dietary supplements, and antibiotics have been explored, but their efficacy remains limited, especially due to the absence of large-scale, high-quality randomized controlled trials (RCTs) to support their clinical application (Mo et al., 2024). For instance, Mediterranean diets and polyphenol dietary supplements have shown potential benefits in long-term interventions (Gutierrez et al., 2021), while antibiotics may have adverse effects on AD by accelerating amyloid plaque deposition (Ternák et al., 2022).

In contrast, TCM, with its holistic approach and multi-target, multi-mechanism advantages, has become a major focus for research on regulating gut microbiota in AD intervention. Its multiple bioactivities—such as anti-inflammatory, immune-regulatory, and neuroprotective effects—have been elucidated with the support of modern microbiomic technologies. From oral administration and gastrointestinal absorption to the metabolism and conversion of active components, TCM can effectively regulate gut microbiota composition and abundance, enhance gut barrier function, reduce bacterial amyloid deposition, inhibit neuroinflammation, and regulate metabolic functions. These mechanisms significantly improve AD-related gut microbiota imbalances, alleviate neurotoxic damage to the central nervous system, and promote neuronal survival and functional recovery. These effects are reflected not only in the synergistic action between the microbiota and the host but also in the conversion of TCM components in microbial metabolism and their downstream functional effects.

However, while evaluating the efficacy of TCM, it is crucial to address its potential safety concerns. The studies included in this review did not report any adverse reactions or toxicity associated with TCM, but this does not imply that TCM is completely safe. The absence of toxicity reports may be attributed to factors such as study design limitations and reporting bias. Previous literature has highlighted that incomplete reporting of adverse events can lead to an underestimation of risks, thus compromising the accurate assessment of treatment safety (Hu J. et al., 2020). For instance, a systematic review revealed that approximately 25.1% of Chinese herbal RCTs did not report any adverse events (Hu J. et al., 2020). Therefore, it is vital to systematically monitor and report potential toxic side effects in future research.

It is also important to emphasize the role of the gut microbiota in drug metabolism, toxicity, and interactions. Increasing evidence indicates that the gut microbiota can influence the activation or inactivation of drugs, thereby modulating their efficacy and toxicity (Feng et al., 2020; Watanabe et al., 2020). A notable example is the chemotherapy drug irinotecan (CPT-11), which is metabolized in the liver into the non-toxic glucuronide conjugate SN-38G. However, in the intestine, bacterial β-glucuronidase hydrolyzes it, releasing the toxic active metabolite SN-38, which causes dose-limiting gastrointestinal toxicity, such as refractory diarrhea (Gao et al., 2022). Similarly, the small intestinal mucosal damage caused by NSAIDs is related to the microbiota: primary bile acids are converted into secondary bile acids by intestinal bacterial enzymes, which are more cytotoxic to the intestinal epithelium. As a result, NSAIDs may alter bile acid toxicity and exacerbate small bowel injury by disrupting the gut microbiota (Wang X. et al., 2021). On the other hand, the gut microbiota’s metabolism of herbal medicine components can also influence the strength of their toxic side effects. For instance, the bis-ester-type diterpenoid alkaloid aconitine, a major toxic component of Aconitum, undergoes dehydration and hydrolysis in the gut under microbiota influence, producing less toxic monomeric ester and lipid alkaloid metabolites, thus reducing its overall toxicity (Yin et al., 2022). Conversely, some herbal components may produce more active and potentially more toxic products after microbial metabolism (Başaran et al., 2022), underscoring the dual regulatory role of the gut microbiota in TCM toxicity.

Furthermore, the combined use of Chinese and Western medicines is common in clinical practice. For instance, Alzheimer’s disease (AD) patients with comorbid metabolic syndrome such as hypertension, coronary heart disease, and diabetes often take both Chinese herbal formulations and Western medications such as antihypertensive and hypoglycemic drugs. In this case, the potential risks of drug interactions deserve special attention. The gut microbiota may play a synergistic or antagonistic role in the mechanisms of combined drug therapy: on the one hand, active components of Chinese medicines and Western drugs may work synergistically through the joint regulation of the microbiota; on the other hand, they may affect each other’s efficacy by competing for or altering metabolic pathways of the microbiota. Such phenomena have been observed in studies on the combined use of hypoglycemic Chinese medicines: Huanglian-containing Chinese medicines with berberine can improve gut microbiota dysbiosis (increasing short-chain fatty acid-producing bacteria and reducing harmful bacteria), enhancing the hypoglycemic effect (Zheng et al., 2020). However, the anthraquinone-containing Chinese herb Da Huang requires intestinal microbiota to convert its conjugated anthraquinone into its free form to exert its effects. Huanglian, by inhibiting this microbiota-mediated transformation, reduces the formation of active components of Da Huang, while Da Huang also partially offsets the effect of Huanglian by affecting the metabolism of Huanglian components in the gut (Zheng et al., 2020). This demonstrates that complex interactions at the microbiota level may occur during the combined use of Chinese and Western medicines, including both synergistic and antagonistic mechanisms.

In conclusion, while no adverse reactions to TCM were observed in this study, the mechanisms discussed suggest that attention should be given to the potential toxicity and interactions between TCM and commonly used Western drugs in treating AD and related comorbidities. Future research should focus on systematically improving the monitoring and reporting of adverse reactions in clinical trials, clarifying the gut microbiota-mediated toxicity of TCM, and ensuring the safety of combined Chinese and Western drug therapies. This is essential to safeguard the safety of medications for AD comorbidity patients, elucidate the role of the gut microbiota in TCM action, and guide safe clinical drug use.

Research has shown that probiotic fermentation can enhance the activity of TCM components, reduce toxicity, and produce new pharmacological effects, providing novel avenues for studying TCM-gut microbiota interactions (Yang et al., 2023). The SHIME^®^ system, which simulates the human gut microbial environment, though still in the exploratory stage, has shown potential in elucidating the mechanisms of action between TCM and gut microbiota (Zhu W. et al., 2024). For example, Falduto M et al. studied the regulatory effects of *C. reticulata* on gut microbiota using the SHIME system, revealing its anti-obesity mechanisms (Falduto et al., 2022). Another promising development is the creation of the MicrobeTCM database, which accelerates the study of TCM and microbiomes. By establishing connections between microbes, botanical drugs, chemical structures, acupuncture points, and diseases, this database can predict relationships between herbal formulas, diseases, microbiota, and target genes (Chen et al., 2024c).

Additionally, gender and age significantly influence the interactions between Alzheimer’s disease (AD) and the gut microbiota. Increasing evidence suggests that, in postmenopausal women, the gut microbiota composition changes significantly, with decreased microbial diversity, which may lead to a reduction in beneficial bacteria and an increase in potentially pathogenic bacteria, resulting in higher intestinal permeability and exacerbated chronic inflammation (Shieh et al., 2020). Estrogen depletion is considered a critical factor, as decreased estrogen levels not only affect neuronal survival and synaptic plasticity but also exacerbate brain inflammation through microbiota-immune pathways. Consequently, clinical observations show that female AD patients tend to experience faster disease progression: compared to male patients, female patients exhibit faster cognitive decline, earlier loss of independence, and more severe disability (Bourzac, 2025). Under similar pathological conditions, female patients initially maintain relatively good cognitive function and brain structural integrity; however, once a “tipping point” is reached, the decline accelerates, leading to rapid hippocampal atrophy (Bourzac, 2025). This may be related to the depletion of female patients’ early neural reserves. Similarly, male patients’ gut microbiota dysbiosis may also drive AD pathology progression through specific immune mechanisms—such as excessive endotoxins (LPS) activating pro-inflammatory pathways, in which glycogen synthase kinase-3β (GSK-3β) is over-activated, promoting tau protein hyperphosphorylation and exacerbating neurofibrillary tangles (Jia Y.-r. et al., 2023). Therefore, when studying the AD-gut axis, gender and age factors must be fully considered: hormonal changes in elderly women and the general decline in microbial diversity in older adults are inflammatory risk factors, while males may contribute to tau pathology through different pathways (e.g., LPS-GSK-3β signaling). Future research should incorporate gender and age stratification to explore the heterogeneous mechanisms of gut microbiota-brain axis regulation in different populations.

Despite the promising potential of TCM in regulating gut microbiota to treat AD, several challenges remain. First, most research is still in the animal model or *in vitro* stage, with a lack of large-scale, high-quality clinical trial data to validate its efficacy and safety. Second, the transformation of TCM interventions faces significant challenges in standardization and regulation. The standardization of Chinese herbal preparations is particularly challenging, as TCM formulas contain multiple active ingredients, and there are considerable variations in the active ingredient content between different batches and manufacturers (Zhang et al., 2012). This variability complicates the replication of studies and challenges dose consistency and quality control. In fact, the lack of standardized drug preparation undermines the reliability of clinical trials and complicates translating research findings into clinical practice. Furthermore, regulatory requirements place higher standards on TCM. Regulatory bodies such as the U.S. FDA and European Medicines Agency (EMA) generally treat TCMs claiming therapeutic efficacy as drugs, requiring them to adhere to the same development processes as conventional new drugs (Liang et al., 2021). This means that Chinese herbal formulas must provide clear evidence of safety and efficacy, including rigorous pharmacological, toxicological evaluations, and phased clinical trials before entering international markets. This process is costly, time-consuming, and especially challenging for formulas with multiple herbal ingredients. Moreover, TCM emphasizes individualized treatment based on syndrome differentiation, which inherently conflicts with the “uniform treatment protocol” required in randomized controlled trials (Fung and Linn, 2015). Balancing TCM’s essence with the need to design reproducible and evaluable clinical research protocols remains a significant challenge. Some progress has been made, including using fingerprinting technologies for quality control and developing realistic placebos for double-blind trials (Balkrishna et al., 2024). Nevertheless, overall, the modernization and transformation of TCM remain exploratory. Addressing challenges in standardization, dose consistency, and regulatory compliance is crucial to facilitating the transition of TCM interventions from experimental research to clinical application. Finally, the complex interactions between TCM, gut microbiota, and AD require a comprehensive research approach.

Limitations of the Literature and Critical Analysis: It is essential to highlight that the quality of the evidence in the literature reviewed here (Tables 1–6) has certain limitations that require careful interpretation. Many studies suffer from small sample sizes, lack rigorous study designs, and are devoid of randomized controlled trials (RCTs), which diminishes the statistical reliability and confidence of their conclusions (Dai et al., 2022). Particularly in the nascent field of “TCM-gut microbiota-AD,” the evidence base remains predominantly rooted in animal models; clinical trial data are only emerging and critically lack support from large-scale, multi-center RCTs. Furthermore, substantial methodological heterogeneity exists across studies, including variations in interventions, follow-up durations, and outcome measures, complicating cross-study comparisons and interpretation. Risks of bias are also concerning, as some studies failed to implement adequate blinding and controls, potentially inflating effect estimates, and publication bias likely favors the reporting of positive results. Consequently, the current literature provides an insufficient evidence base, rendering many findings, particularly regarding correlations, exploratory in nature; causal inferences and generalizability to human populations require rigorous validation through higher-quality studies (Dai et al., 2022; Liu M. et al., 2024).

**TABLE 6:** Critical appraisal of included studies.

TCM intervention	Application dose	Intervention time	Model type	Positive controls	Strain identification method	References
Banxia Xiexin Decoction	6 g/kg/day [twice of adult equivalent doses (70 kg)]	3 months	*In vivo* experiments (APP/PS1 mice)	Liraglutide Injection [Novo Nordisk (China) Pharmaceuticals Co., Ltd., lot No. S20160004, 25 nmol/kg]	16S rRNA gene sequencing	Gao et al. (2023)
Chaihu Shugan San	2.1 g/kg/day and 4.2 g/kg/day	8 weeks	*In vivo* experiments (SAMP8 mice)	Donepezil (1 mg/kg/day)	16S rRNA gene sequencing	Li et al. (2023c)
Qi-Fu-Yin	3.1 g/kg/day, 6.2 g/kg/day, and 12.4 g/kg/day	195 days	*In vivo* experiments (APP/PS1 mice)	Donepezil (1.0 mg/kg/d) +Memantine (2.8 mg/kg/d)	Metagenomic sequencing	Xiao et al. (2022)
CA-30, an oligosaccharide fraction derived from Liuwei Dihuang decoction	12.24 mg/10g/day	199 days	*In vivo* experiments (SAMP8 mice)	-(deionized water)	Metagenomic sequencing	Wang et al. (2019a)
Epigallocatechin-3-gallate (EGCG)	5 mg/kg/day, 15 mg/kg/day, and 45 mg/kg/day	8 weeks	*In vivo* experiments (HFD (HFOVX)-fed estrogen-deficient mice)	-(Ovariectomized (OVX) mice fed an HFD)	16S rRNA gene sequencing	Qu et al. (2022b)
Ginsenoside Rg1	7.5 mg/kg/day, 15 mg/kg/day, and 30 mg/kg/day	8 weeks	*In vivo* experiments (D-gal + Aβ25-35 induced tree shrews)	Donepezil (3 mg/kg/day)	16S rRNA gene sequencing	Wang et al. (2020a)
Ginsenoside Rg1	30 mg/kg/day	6 weeks	*In vivo* experiments (D-gal + Aβ25-35 induced tree shrews)	-(saline)	16S rRNA gene sequencing	Guo et al. (2021)
Silibinin and Silymarin	100 mg/kg/day	15 days	*In vivo* experiments (APP/PS1 mice)	-	16S rRNA gene sequencing	Shen et al. (2019)
Quercetin	AIN-93G diet supplemented with 0.08% quercetin	21 days	*In vivo* experiments (Aged ICR mice induced by short-term dietary intake of advanced glycation end products)	-(AIN-93G diet)	16S rRNA gene sequencing	Yang et al. (2020)
Quercetin	modified AIN-93G diet (i.e., the VD level was cut into half, VD level = 500 IU kg^−1^ diet) supplemented with0.08% quercetin (i.e., 80 mg quercetin dissolved in 100 g food) *ad libitum*; AIN-93G diet (VD level = 1000 IU kg^−1^) supplemented with 0.08% quercetin *ad libitum*; AIN-93G diet (VD level = 1000 IU kg^−1^) supplemented with 0.08% quercetin *ad libitum* and extra 1000 IU VD3 by intramuscularly injection every 2 weeks	20 weeks	*In vivo* experiments (APP/PS1 mice)	-(AIN-93G diet)	16S rRNA gene sequencing	Lv et al. (2018)
Total saikosaponins	20 mg/kg/day, 40 mg/kg/day, and 80 mg/kg/day	30 days	*In vivo* experiments (APP/PS1 mice)	Donepezil (5 mg/kg/day)	16S rRNA gene sequencing	Li et al. (2022b)
2H-gentiopicroside	1 mg/kg/day, 3 mg/kg/day, and 10 mg/kg/day	9 weeks	*In vivo* experiments (D-galactose-induced AD mice)	0.85% stroke-physiological saline solution	16S rRNA gene sequencing	Wang et al. (2023a)
Ginkgolide B	0.1% Ginkgolide B	4 weeks	*In vivo* experiments (Mice induced by D-galactose and aluminum chloride)	-(saline)	16S rRNA gene sequencing	Liu et al. (2021)
Gastrodin	100 mg/kg/day	4 weeks	*In vivo* experiments (APP/PS1 mice)	-(saline)	16S rRNA gene sequencing	Zhang et al. (2023b)
Bushen Yinao pill	8–12 pills twice daily	6 months	*In vivo* experiments (AD patients)	Donepezil hydrochloride at a dosage of 5 mg once daily, and Piracetam at a dosage of 0.8 g three times daily	Not mentioned	Wang et al. (2025)
Kai-xin-san	10 g/kg/day	8 weeks	*In vivo* experiments (D-gal + Aβ25-35 induced rats)	Huperzine A (30 μg/kg)	16S rRNA gene sequencing FMT	Wang et al. (2024a)
Jiedu Yizhi Formula	10.536 g/kg/d and 20.268 g/kg/d	8 weeks	*In vivo* experiments (APP/PS1 mice)	Donepezil hydrochloride (0.45 g/kg)	16S rRNA gene sequencing	Zhang et al. (2023a)
*Tetragonia tetragonioides*	0.5% of TTK-E extract	47 days	*In vivo* experiments (Aβ25-35-induced high-fat diet in AD rats)	-(0.5% dextrin)	16S rRNA gene sequencing	Kim et al. (2020)
Gegen Qinlian tablets	130 g/kg/day and 324 g/kg/day	14 weeks	*In vivo* experiments (AD rats induced by Aβ1-42)	-(0.3% CMC Na solution)	16S rRNA gene sequencing	Wang et al. (2024b)
Scutellarin	20 mg/kg/day and 100 mg/kg/day	8 weeks	*In vivo* experiments (APP/PS1 mice)	-(0.1% sodium carboxymethyl cellulose)	16S rRNA gene sequencing	Zhang et al. (2022c)
Patchouli alcohol	25 mg/kg/day and 50 mg/kg/day	4 months	*In vivo* experiments (TgCRND8 transgenic AD mouse)	Donepezil (5 mg/kg)	16S rRNA gene sequencing FMT	Xu et al. (2023)
Curcumin	Dietary intervention:curcumin-supplemented (4 g/kg)	14 weeks	*In vivo* experiments (3xTg-AD mice)	-	16S rRNA gene sequencing	Lamichhane et al. (2024)
Modified Huang-Lian-Jie-Du Decoction	3.5 g/kg/day and 7 g/kg/day	3 weeks	*In vivo* experiments (Aβ1-42-induced C57BL/6 mice)	Donepezi (2 mg/kg/day)	16S rRNA gene sequencing	Liu et al. (2019)
Hawthorn flavonoid	100 mg/kg/day, 200 mg/kg/day, and 400 mg/kg/day	35 days	*In vivo* experiments (AD mice induced by D-galactose and aluminum chloride)	Donepezi (1 mg/kg/day)	16S rRNA gene sequencing	Zhang et al. (2022b)
Quercetin-3-O-Glucuronide	50 mg/kg/day (vivo) 20 μM(vitro)	4 weeks	*In vivo* experiments (AD mice induced by Aβ1-42); *In vitro* SH-SY5Y cells	-	16S rRNA gene sequencing	Xu et al. (2021)
*Schisandra chinensis polysaccharide*	20 mg/kg/day (Previous month) and 50 mg/kg/day (Next month)	2 months	*In vivo* experiments (AD rat model induced by Aβ25-35)	-(saline)	16S rRNA gene sequencing	Fu et al. (2023)
Walnut-Derived Peptide (PW5)	80 mg/kg/day and 400 mg/kg/day	12 weeks	*In vivo* experiments (APP/PS1 mice)	-(saline)	16S rRNA gene sequencing	Wang et al. (2019b)
Oligosaccharides from Morinda officinalis	100 mg/kg/day	6 months	*In vivo* experiments (APP/PS1 mice)	-(distilled water)	16S rRNA gene sequencing	Xin et al. (2019)
Danggui Shaoyao San	24 g/kg/day	14 days	*In vivo* experiments (STZ-induced AD rats)	-(saline)	16S rRNA gene sequencing	Jin et al. (2023)
GuanXinNing Tablet	250 mg/kg/day	12 weeks	*In vivo* experiments (An AD model rabbit fed a 2% cholesterol diet)	-	16S rRNA gene sequencing	Zhang et al. (2021a)
Rich in phenylpropanoids-polyacetylenes and polysaccharides from *Codonopsis Radix*	EPP (0.2 g/kg/day, 0.4 g/kg/day, and 0.6 g/kg/day), and POL (0.3 g/kg/day, 0.6 g/kg/day, and 0.9 g/kg/day)	16 days	*In vivo* experiments (SCOP-induced AD mouse model)	Donepezil (5 mg/kg)	16S rRNA gene sequencing	Xie et al. (2024)
Danggui Shaoyao San	24 g/kg/day	14 days	*In vivo* experiments (STZ-induced AD rats)	-(saline)	16S rRNA gene sequencing	He et al. (2023)
*Rheum tanguticum*	0.91 g/kg/day	60 Days	*In vivo* experiments (APP/PS1 mice)	Donepezil (1.5 mg/kg)	16S rRNA gene sequencing	Gao et al. (2021)
Baicalein	25 mg/kg/day and 50 mg/kg/day	14 days	*In vivo* experiments (APP/PS1 mice)	-	Metagenomic sequencing	Shi et al. (2023)
*Poria cocos*	1.2 g/kg/day	3 months	*In vivo* experiments (APP/PS1 mice)	-(saline)	16S rRNA gene sequencing	Sun et al. (2021)
Schisandrin	10 mg/kg/day	8 weeks	*In vivo* experiments (D-gal + Aβ25-35 induced rats)	-(saline)	16S rRNA gene sequencing	Zhang et al. (2022a)
Icariin	100 g/kg/day	100 days	*In vivo* experiments (APP/PS1 mice)	-(saline)	16S rRNA gene sequencing	Liu et al. (2023)
Icariin and Panax notoginseng Saponins	80 mg/kg/d ICA and 150 mg/kg/d PNS	4 weeks	*In vivo* experiments (APP/PS1 mice)	Donepezil (1.30 mg/kg/d)	16S rRNA gene sequencing	Zhang et al. (2020)
Ginkgo biloba Extract	100 mg/kg/day	2 months	*In vivo* experiments (APP/PS1 mice)	-(saline)	Metagenomic sequencing	Yu et al. (2023)
P-coumaric acid	20 mg/kg/day and 40 mg/kg/day	28 days	*In vivo* experiments (AD mice induced by Aβ25-35)	Huperzine A (0.2 mg/kg/day)	16S rRNA gene sequencing	Cao et al. (2023)
Korean Red Ginseng	30 mg/kg/day and 100 mg/kg/day	2 weeks	*In vivo* experiments (Tg2576 heterozygous transgenic mice)	-(distilled water)	16S rRNA gene sequencing	Lee et al. (2022b)
*Polygonatum sibiricum polysaccharides*	30 mg/kg/day	3 months	*In vivo* experiments (5xFAD mice)	-	16S rRNA gene sequencing	Luo et al. (2022)
Sinomenine	40 mg/kg/day and 80 mg/kg/day		*In vivo* experiments (SCOP-induced AD mouse model)	Donepezil (3 mg/kg/day)	16S rRNA gene sequencing	Ni et al. (2024)
Berberine	100 mg/kg/day and 200 mg/kg/day	28 days	*In vivo* experiments (5xFAD mice)	-(saline)	16S rRNA gene sequencing	Sun et al. (2024)
Oligosaccharides From Morinda officinalis	50 mg/kg/day and 100 mg/kg/day	6 months	*In vivo* experiments (APP/PS1 mice)	-(saline)	16S rRNA gene sequencing	Xin et al. (2018)
Fructooligosaccharides from Morinda officinalis	50 mg/kg/day and 100 mg/kg/day	8 weeks	*In vivo* experiments (D-galactose and Aβ1-42-induced AD rats)	-(saline)	16S rRNA gene sequencing	Chen et al. (2017)
Gastrodin	3 mg/kg/days, 90 mg/kg/day, and 210 mg/kg/day	9 weeks	*In vivo* experiments (D-galactose-induced AD mice)	Donepezil (3 mg/kg/day)	16S rRNA gene sequencing	Fasina et al. (2022)

Limitations of the research and future outlook: Building upon the critical analysis of the literature, the current state of research in “TCM-gut microbiota-AD” faces several pivotal limitations that define crucial avenues for future investigation.

- Unaddressed Population Heterogeneity: Research into the influence of gender and age differences on TCM interventions targeting the gut microbiota for AD remains a significant knowledge gap, limiting the personalization and optimization of therapeutic strategies.
- Translational Chasm and Weak Clinical Validation: The severe paucity of robust clinical evidence, primarily due to the lack of high-quality RCTs as identified in the literature, impedes the conclusive establishment of TCM’s efficacy and applicability in modulating the gut microbiota for AD management in humans. Bridging the gap between promising preclinical findings and clinical utility is paramount.
- Persistent TCM Methodological Hurdles: Inherent complexities in TCM research, including unclear bioactive components, poorly understood multi-component synergistic mechanisms, and challenges in ensuring reliable experimental interventions and placebo controls, continue to pose substantial obstacles, raising concerns about the reproducibility of findings and hindering mechanistic understanding.
- Insufficient Long-Term Perspective and Safety Assessment: Many existing studies feature inadequate follow-up durations, preventing the evaluation of long-term effects on AD progression. Moreover, comprehensive monitoring of dynamic changes in the gut microbiota over time and systematic assessment of long-term safety profiles of interventions are frequently overlooked.

Moving forward, we recommend prioritizing research in the following areas.

(1) Conduct High-Quality Clinical Trials: Design multi-center, large-sample RCTs to validate existing potential efficacy signals. For example, some TCM formulas that have performed well in animal studies could progress from Phase I to II/III clinical trials, ensuring their safety. Study designs should incorporate gender and age variations, investigate individual differences, and utilize rigorous blinding and objective measures to assess efficacy. Adverse reactions should be systematically monitored and reported, with particular attention to toxicity and drug interaction risks, especially for polypharmacy in AD patients.
(2) Strengthen Standardization and Mechanism Studies for TCM: Employ modern analytical techniques (e.g., chemical fingerprinting, mass spectrometry metabolomics) to standardize TCM formulas and identify key active ingredients and their metabolites. Utilizing network pharmacology in combination with metagenomics, transcriptomics, and metabolomics, further elucidate the multi-target mechanisms through which TCM regulates the gut-brain axis. Identifying microbiota changes and metabolic pathways directly linked to AD pathology improvement will help clarify the molecular mechanisms of TCM, including its interactions with the microbiota, and provide a foundation for dosage optimization and drug development.
(3) Focus on Long-Term Safety and Efficacy Follow-Up: Future research should extend follow-up periods to evaluate the long-term impact of gut microbiota modulation on cognitive function and neuroimaging indicators in AD patients, as well as the safety and tolerance of repeated medication use. Special attention should be given to TCMs with potentially toxic components (e.g., Tripterygium wilfordii, Aconitum), with systematic animal and clinical studies conducted to define safe usage ranges and closely monitor the role of the gut microbiota.
(4) Promote Interdisciplinary Collaboration and Translation: Integrate expertise from neurobiology, microbiome research, pharmacy, toxicology, clinical medicine, and regulatory science to jointly explore the “gut microbiota-TCM-AD” complex system. In basic research, gene-editing models could be used to verify the causal role of specific microbiota or metabolites in AD and their interactions with TCM components. By utilizing artificial intelligence and organoid models, experimental designs and data integration can be optimized, improving research efficiency and data reliability. Clinically, consider conducting comparative studies on integrated TCM and Western medicine therapies to identify synergistic treatment options. Additionally, strategies should be developed to address the standardization and regulatory compliance challenges faced by TCM.

In conclusion, research on the regulation of the gut microbiota by TCM in the prevention and treatment of AD is still in its early stages. Challenges remain regarding limited evidence, unclear mechanisms, and safety and translational application. However, with the advancement of the above priority research directions, particularly solving safety concerns, strengthening standardization, conducting high-quality clinical trials, and focusing on individual differences (gender, age), we expect to obtain higher-level evidence to confirm efficacy and overcome translation bottlenecks. This will lay a solid foundation for better integrating TCM into the modern evidence-based medical system and opening new avenues for AD prevention and treatment.
